# Vebreltinib for Previously Treated Astrocytoma, *IDH*-Mutant, Grade 4, and Glioblastoma, *IDH* Wild-Type with *PTPRZ1–MET* Fusion Gene: A Multicenter, Phase III Randomized, Open-Label Trial

**DOI:** 10.34133/cancomm.0019

**Published:** 2026-03-11

**Authors:** Zhaoshi Bao, Yake Xue, Yanhui Liu, Shouwei Li, Liang Wang, Yan Qu, Yonggao Mou, Rutong Yu, Jinsong Wu, Yu Yao, Kai Shu, Guangyuan Hu, Linbo Cai, Wenbin Li, Xiaoguang Qiu, Yunqian Li, Lei Zhang, Songtao Qi, Ying Ji, Chunxiao Ma, Wenbin Ma, Gang Li, Rongjie Tao, Chongran Sun, Ligang Chen, Sheng-Qing Lv, Peng Liang, Hao Pan, Woo Yat Ming Peter, Chan Tat Ming Danny, Qing Mao, Xinting Wei, Tao Jiang

**Affiliations:** ^1^ Beijing Neurosurgical Institute, Beijing, P. R. China.; ^2^ Department of Neurosurgery, Beijing Tiantan Hospital, Capital Medical University, Beijing, P. R. China.; ^3^ Department of Neurosurgery, The First Affiliated Hospital of Zhengzhou University, Zhengzhou, Henan, P. R. China.; ^4^ Department of Neurosurgery, West China Hospital, Sichuan University, Chengdu, Sichuan, P. R. China.; ^5^ Department of Neurosurgery, Sanbo Brain Hospital, Capital Medical University, Beijing, P. R. China.; ^6^ Department of Neurosurgery, Tangdu Hospital, Fourth Military Medical University, Xi’an, Shanxi, P. R. China.; ^7^ Department of Neurosurgery/Neuro-oncology, State Key Laboratory of Oncology in South China, Guangdong Provincial Clinical Research Center for Cancer, Sun Yat-sen University Cancer Center, Guangzhou, Guangdong, P. R. China.; ^8^ Department of Neurosurgery, Affiliated Hospital of Xuzhou Medical University, Xuzhou, Jiangsu, P. R. China.; ^9^ Department of Neurosurgery, Huashan Hospital, Fudan University, Shanghai, P. R. China.; ^10^ Department of Neurosurgery, Tongji Hospital, Tongji Medical College of Huazhong University of Science and Technology, Wuhan, Hubei, P. R. China.; ^11^ Department of Neurosurgery, Guangdong Sanjiu Brain Hospital, Guangzhou, Guangdong, P. R. China.; ^12^ Department of Neuro-oncology, Beijing Tiantan Hospital, Capital Medical University, Beijing, P. R. China.; ^13^ Department of Radiation Oncology, Beijing Tiantan Hospital, Capital Medical University, Beijing, P. R. China.; ^14^ Department of Neurosurgery, The First Hospital of Jilin University, Changchun, Jilin, P. R. China.; ^15^ Department of Neurosurgery, Shengjing Hospital of China Medical University, Shenyang, Liaoning, P. R. China.; ^16^ Department of Neurosurgery, Nanfang Hospital, Southern Medical University, Guangzhou, Guangdong, P. R. China.; ^17^ Department of Neurosurgery, The First Affiliated Hospital of University of Science and Technology of China, Division of Life Sciences and Medicine, University of Science and Technology of China, Hefei, Anhui, P. R. China.; ^18^ Department of Neurosurgery, Henan Provincial People’s Hospital, People’s Hospital of Zhengzhou University, Zhengzhou, Henan, P. R. China.; ^19^ Department of Neurosurgery, Peking Union Medical College Hospital, Beijing, P. R. China.; ^20^ Department of Neurosurgery, Qilu Hospital of Shandong University, Jinan, Shandong, P. R. China.; ^21^ Department of Neurosurgery, Shandong Tumor Hospital, Jinan, Shandong, P. R. China.; ^22^ Department of Neurosurgery, The Second Affiliated Hospital, Zhejiang University School of Medicine, Hangzhou, Zhejiang, P. R. China.; ^23^ Department of Neurosurgery, The Affiliated Hospital of Southwest Medical University, Luzhou, Sichuan, P. R. China.; ^24^ Department of Neurosurgery, Xinqiao Hospital, Third Military Medical University (Army Medical University), Chongqing, P. R. China.; ^25^ Department of Neurosurgery, Harbin Medical University Cancer Hospital, Harbin, Heilongjiang, P. R. China.; ^26^ Department of Neurosurgery, General Hospital of Eastern Theater Command of Chinese People’s Liberation Army, Nanjing, Jiangsu, P. R. China.; ^27^ Department of Neurosurgery, The Affiliated BenQ Hospital of Nanjing Medical University, Nanjing, Jiangsu, P. R. China.; ^28^ The Otto Wong Brain Tumour Centre, Division of Neurosurgery, Department of Surgery, The Chinese University of Hong Kong, New Territories, Hong Kong, P. R. China.

## Abstract

**Background:** High-grade gliomas, including isocitrate dehydrogenase (*IDH*)-mutant astrocytoma and *IDH* wild-type glioblastoma, have a poor prognosis and limited treatment options. The *PTPRZ1–MET* (*ZM*) fusion gene is a potential therapeutic target. This study evaluated vebreltinib, a highly selective, adenosine-triphosphate-competitive inhibitor of the mesenchymal–epithelial transition factor (*MET*), in patients with *ZM*-fusion-positive glioma. **Methods:** In this multicenter, open-label *ZM* FUsion GENe (FUGEN) trial, patients with previously treated astrocytoma, *IDH*-mutant, grade 4, or glioblastoma, *IDH* wild-type, harboring the *ZM* fusion were randomized in a 1:1 ratio to receive vebreltinib (300 mg orally twice daily) or control treatment (temozolomide or cisplatin plus etoposide) in 28-d cycles. The primary end point was overall survival (OS). Key secondary end points included progression-free survival (PFS), objective response rate (ORR), and safety analyses. **Results:** Eighty-one patients (42 in the vebreltinib group and 39 in the control group) were included in the full analysis set. As of 2023 April 1, the median follow-up duration was 5.9 (range, 0.8 to 44.7) months in the vebreltinib group and 3.4 (range, 0.5 to 40.5) months in the control group. Median OS was significantly longer in the vebreltinib group than in the control group (6.3 months versus 3.4 months; hazard ratio [HR], 0.52; 95% confidence interval [CI], 0.32 to 0.85; stratified log-rank *P* = 0.007). In the *IDH*-mutant subgroup, median OS was 7.7 months in the vebreltinib group and 3.3 months in the control group (HR, 0.48; 95% CI, 0.28 to 0.80; stratified log-rank *P* = 0.005). Among patients with a baseline tumor diameter of ≤3.0 cm, median OS was 32.5 months in the vebreltinib group versus 4.2 months in the control group (HR, 0.27; 95% CI, 0.07 to 1.06; stratified log-rank *P* = 0.046). Median PFS was also longer in the vebreltinib group (1.9 months versus 1.1 months; HR, 0.54; 95% CI, 0.33 to 0.88; stratified log-rank *P* = 0.012). The ORR was 9.5% with vebreltinib and 2.6% with control treatment. The incidence of grade ≥3 adverse events was comparable between groups, and no treatment-related deaths were reported. **Conclusion:** Vebreltinib significantly improved OS in patients with previously treated high-grade glioma harboring the *ZM* fusion, particularly in the subgroup with *IDH*-mutant astrocytoma, and the safety profile was manageable. **Trial registration:** This study was registered with the Chinese Drug Clinical Trial Registry (ChinaDrugTrials.org.cn) under the identifier, CTR20181664 (registration date: 2018 September 19).

## Introduction

Gliomas represent the most common and lethal malignant primary tumors of the central nervous system (CNS), accounting for the majority of CNS-tumor-related deaths worldwide [1,2]. These tumors are characterized by marked intrinsic heterogeneity. While isocitrate dehydrogenase (*IDH*)-mutant gliomas often progress from lower- to higher-grade malignancies, *IDH* wild-type glioblastomas typically arise de novo as aggressive, high-grade tumors without a recognized precursor lesion [3,4]. Despite advances in molecular characterization, high-grade gliomas harboring *IDH* mutations continue to pose a therapeutic challenge, with few effective targeted therapies available [5]. The current standard of care for glioblastoma involves maximal safe surgical resection, followed by radiotherapy and temozolomide-based chemotherapy, providing limited clinical efficacy, with a median overall survival (OS) of about 15 months despite aggressive multimodal therapy [6]. For first-line treatment of *IDH*-mutant grade 4 astrocytoma, the standard of care includes radiotherapy, temozolomide, and alternating electric fields, either alone or in combination, depending on the patient’s Karnofsky performance status (KPS) [7]. The unmet clinical need is also pressing for recurrent disease, where therapeutic options are severely limited and prognosis remains dismal [8]. The discovery of the *PTPRZ1–MET* (*ZM*) fusion gene in approximately 15% of secondary glioblastomas (now reclassified under the 5th edition of the World Health Organization classification of CNS tumors [WHO CNS5] as astrocytoma, *IDH*-mutant, grade 4) has provided important insights into glioma progression and therapeutic vulnerabilities [9]. The *ZM* fusion has been implicated in promoting malignant transformation and resistance to standard therapies and is now recognized as a potential molecular target in glioma [10–12]. Initial preclinical studies demonstrated the oncogenic role of the *ZM* fusion in glioma biology, while subsequent investigations identified mesenchymal–epithelial transition factor (*MET*) signaling as a rational therapeutic target in this context [9,13,14].

Vebreltinib, a next-generation adenosine-triphosphate-competitive inhibitor of the *MET*, exhibits a favorable pharmacologic profile, including enhanced brain penetration, high apparent permeability, and a low efflux ratio [15]. These features render vebreltinib a compelling candidate for therapeutic intervention in gliomas harboring the *ZM* fusion and support its clinical evaluation. In a phase I, open-label clinical study [15] involving patients with recurrent high-grade gliomas harboring the *ZM* fusion, vebreltinib demonstrated encouraging preliminary efficacy. Among 6 patients with secondary glioblastoma harboring the *ZM* fusion, 2 achieved partial responses, and 2 had stable disease. Importantly, no serious adverse events (SAEs) were reported [15]. These early findings provided the rationale for investigating the role of vebreltinib in randomized clinical trials.

In this multicenter, open-label, *ZM* FUsion GENe (FUGEN) trial, we evaluated the clinical benefit of vebreltinib in patients with previously treated astrocytoma, *IDH*-mutant, grade 4, or glioblastoma, *IDH* wild-type, harboring the *ZM* fusion.

## Methods

### Study design and patients

This multicenter, randomized, open-label trial (FUGEN) evaluated the efficacy and safety of vebreltinib in patients aged 18 to 65 years with previously treated, histologically confirmed astrocytoma, *IDH*-mutant, grade 4, or glioblastoma, *IDH* wild-type, with *ZM* gene fusion, in accordance with WHO CNS5 [16]. Eligibility for the study required molecular confirmation of the *ZM* fusion gene in the latest surgical sample. The presence of the *ZM* fusion was detected using polymerase chain reaction, as previously described [13]. Briefly, primers flanking the fusion breakpoint were used (forward, 5′-CCGTCTGGAAATGCGAATCCTAAA-3′; reverse, 5′-CAGGCCCAGTCTTGTACTCAGCAA-3′), and analyses were performed in a central laboratory. Genetic testing was performed centrally at AmoyDx (Xiamen, China). Additional inclusion criteria included a KPS score of 60 or higher, a life expectancy of more than 3 months, prior radiotherapy and/or temozolomide treatment with evidence of tumor recurrence, and adequate hepatic and renal function. Patients were eligible if they had received antibody-based anticancer therapy (e.g., bevacizumab) more than 30 d prior to enrollment. Tumor recurrence was determined according to the Response Assessment in Neuro-Oncology (RANO) criteria, which incorporates both clinical and radiological factors [17,18]. The final assessment was made by local, multidisciplinary neuro-oncology teams based on comprehensive clinical judgment, which included an evaluation of serial dynamic magnetic resonance imaging (MRI) trends, clinical neurological status, and corticosteroid dosage. At the time of inclusion, the estimated life expectancy of patients was evaluated on the basis of the median OS of 127 d, as reported in a previous study [9], and was further determined with the Chinese Glioma Genome Atlas database (https://www.cgga.org.cn/). Using this tool, clinical information, such as gene mutation status and tumor subtype, was entered, and survival curves were plotted accordingly.

Patients were excluded if they had received prior *MET* inhibitors or hepatocyte growth factor (*HGF*)-targeted therapies. The use of glucocorticoids was permitted only if discontinued or maintained at a stable or tapering dose for at least 5 d before enrollment. Complete inclusion and exclusion criteria are provided in the Supplementary Materials. The trial was conducted in accordance with the Declaration of Helsinki and applicable Good Clinical Practice guidelines. Written informed consent was obtained from all patients or their legal representatives, and ethical approval was secured from the institutional review board or independent ethics committee at each participating site (see the Supplementary Materials**)**. This study was registered with the Chinese Drug Clinical Trial Registry (ChinaDrugTrials.org.cn) under the identifier CTR20181664.

### Procedures

Patients meeting the eligibility criteria were stratified according to KPS scores (≥60 and <80 versus ≥80) and randomly assigned in a 1:1 ratio to receive either vebreltinib or standard therapy (control group) using a centralized, interactive web-based response system. Patients in the vebreltinib group received 300 mg orally twice daily. Those in the control group received either temozolomide at a dose of 100 to 150 mg/m^2^ daily for 7 d, followed by a 7-d interval, or a combination regimen of cisplatin (80 to 100 mg/m^2^) and etoposide (100 mg/m^2^) for 3 d, repeated every 28 d. The dose of 300 mg orally twice daily was selected on the basis of data from a phase I clinical trial [15], which showed a favorable safety profile, preliminary efficacy, and linear pharmacokinetics across doses from 50 to 300 mg. At 300 mg twice daily, vebreltinib achieved the highest concentration in cerebrospinal fluid, correlating with the peak plasma exposure, supporting its efficacy and safety at this dose. The use of the following treatments was prohibited throughout the study: any other class of chemotherapeutic agents, immunotherapy or immunosuppressive therapy (except for glucocorticoids), other investigational drugs, craniotomy, interstitial therapy for tumors, any form of radiation therapy, and strong inhibitors or inducers of cytochrome P450 3A4 (CYP3A4) enzymes. Following disease progression, continuation of the study treatment, either as monotherapy or in combination with other agents (e.g., bevacizumab), was permitted if deemed clinically beneficial by the investigator.

Imaging assessments were conducted according to a standardized protocol described previously [19], with MRI performed at baseline and at weeks 4, 8, 12, 16, 24, 36, and 48. Treatment continued for up to 48 weeks or until the occurrence of intolerable toxicity, radiographic or clinical disease progression, or death, whichever occurred first.

### End points and assessment

The primary end point was OS, defined as the time from randomization to death from any cause; patients who were still alive at the end of the study were censored at the date of last contact. Key secondary end points included progression-free survival (PFS), defined as the time from randomization to the first documented radiographic disease progression or death from any cause, whichever occurred first, and objective response rate (ORR), defined as the proportion of patients achieving a complete or partial response per the RANO criteria. Other secondary end points included changes in KPS scores and health-related quality of life, as assessed by the European Organisation for Research and Treatment of Cancer Quality of Life Questionnaire Core 30 (EORTC QLQ-C30) and Quality of Life Questionnaire Brain Cancer Module 20 (QLQ-BN20). Safety assessments included physical examinations (with neurologic evaluations), KPS scores, vital signs, 12-lead electrocardiograms, clinical laboratory tests (hematologic, biochemical, and coagulation parameters), and the incidence and severity of adverse events (AEs), which were graded according to the National Cancer Institute Common Terminology Criteria for Adverse Events, version 5.0. Efficacy and safety were assessed at the end of weeks 4, 8, 12, 16, 24, 36, and 48. A safety follow-up visit was conducted 4 weeks after the final dose, and survival status was monitored every 4 weeks thereafter.

### Statistical analysis

Assuming a median OS of 4 months in the control group, a sample size of 74 patients (including 70 death events) could detect a 4-month improvement in the vebreltinib group with 80% power, using a one-sided log-rank test at a significance level of 0.025. Accounting for a 10% dropout rate, the final planned enrollment was 84 patients. Efficacy analyses were performed in the full analysis set (FAS), which included all patients who met the following criteria: (a) histologically confirmed astrocytoma, *IDH*-mutant, grade 4, or glioblastoma, *IDH* wild-type, in accordance with the WHO CNS5 [16]; (b) molecular confirmation of a *ZM* fusion gene; (c) recurrent disease following prior radiotherapy and temozolomide, or ineligibility for radiotherapy, or intolerance to temozolomide; and (d) receipt of at least one dose of the study drug with at least one postbaseline assessment. To provide a more conservative estimate, efficacy and survival analyses were further conducted in the intention-to-treat (ITT) population, which included all randomized patients. Safety analyses were conducted in the safety set (SS), comprising all patients who received at least one dose of the assigned treatment and had available posttreatment safety data. Categorical variables were summarized as frequencies and percentages, while continuous variables were described using mean, SD, medians, interquartile range (IQR), and range. OS and PFS were estimated using the Kaplan–Meier method with 2-sided 95% confidence intervals (CIs). Comparisons between treatment groups for OS and PFS were performed using stratified log-rank tests based on the randomization stratification factors. Hazard ratios (HRs) and corresponding CIs were derived from stratified Cox proportional hazards models. Prespecified subgroup analyses were conducted for both OS and PFS according to KPS score, age, sex, *IDH* status, history of surgical resection, bevacizumab use, and tumor size. In addition, a post hoc subgroup analysis was performed to explore the association between extent of surgical resection (gross total resection, subtotal resection, or partial resection), *O*^6^-methylguanine-DNA methyltransferase (*MGMT*) promoter methylation status, and survival outcomes. The ORR and its 95% CI were calculated for each group, along with the distribution of best overall responses.

## Results

### Patient characteristics

**Fig. 1.**
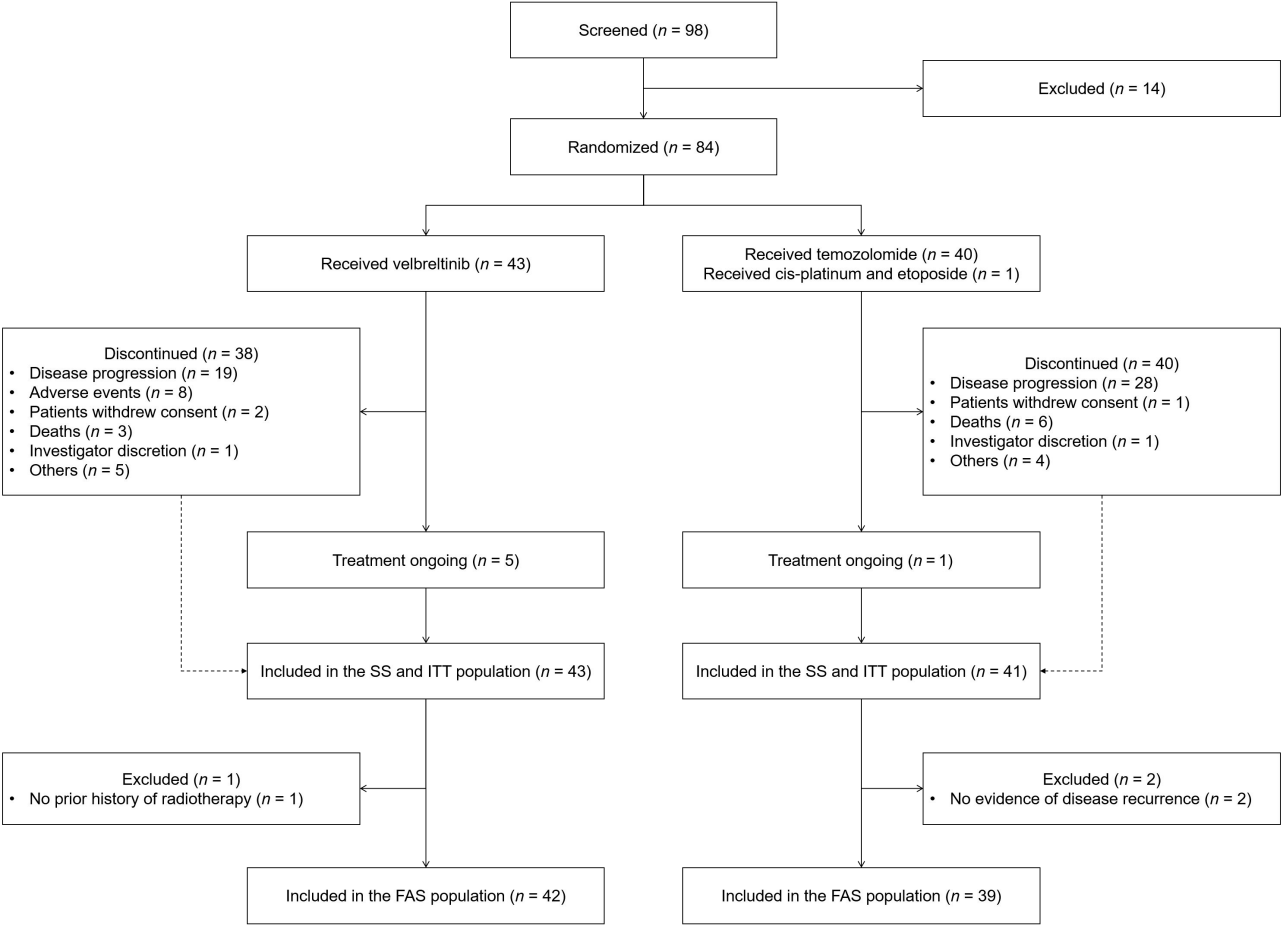
Flowchart of patient screening and randomization. In the vebreltinib group, the reported discontinuations due to AEs included cases triggered by symptomatic worsening. Subsequent blinded independent radiographic review determined that 5 of the 8 patients who discontinued treatment for this reason had concurrent disease progression. FAS included all patients who met the following criteria: (a) histologically confirmed astrocytoma, *IDH*-mutant, grade 4, or glioblastoma, *IDH* wild-type; (b) molecular confirmation of a *ZM* fusion gene; (c) recurrent disease following prior radiotherapy and temozolomide, or ineligibility for radiotherapy, or intolerance to temozolomide; and (d) receipt of at least one dose of the study drug with at least one postbaseline assessment. ITT population included all randomized patients. SS included all patients who received at least one dose of the assigned treatment and had available posttreatment safety data. SS, safety set; ITT, intention-to-treat; FAS, full analysis set.

Between July 2018 and August 2020, a total of 98 patients were screened, of whom 84 were enrolled across 18 clinical centers. Of these, 43 were randomly assigned to the vebreltinib group and 41 to the control group (Fig. 1). Within the control arm, 40 patients received temozolomide, and 1 patient received a combination of cisplatin and etoposide. A total of 81 patients (42 in the vebreltinib group and 39 in the control group) were included in the FAS, and all 84 patients were included in the SS.

Baseline characteristics were well balanced between groups (Table 1). The median age was 41.0 (IQR, 35.0 to 48.0) years in the vebreltinib group and 40.0 (IQR, 32.0 to 49.0) years in the control group. Males represented 22 (52.4%) and 22 (56.4%) of the vebreltinib and control groups, respectively. The median KPS score in both groups was 70 (IQR, 60 to 80). Tumor diameter at baseline exceeded 3.0 cm in 31 (73.8%) of patients in the vebreltinib group and 26 (66.7%) in the control group. The median interval from initial diagnosis was 35.5 (IQR, 20.6 to 67.0) months in the vebreltinib group and 51.1 (IQR, 25.0 to 65.4) months in the control group. Astrocytoma, *IDH*-mutant, grade 4 was present in 39 (92.9%) and 34 (87.2%) of patients in the vebreltinib and control groups, respectively. All patients had received prior surgery, radiotherapy, and chemotherapy. *MGMT* promoter methylation status was available for a subset of patients at baseline (Table 1). In the vebreltinib group, 8 patients (19.0%) had *MGMT* promoter-methylated tumors, and 11 (26.2%) had *MGMT* promoter-unmethylated tumors; *MGMT* promoter methylation status was missing in 23 patients (54.8%). In the control group, 11 patients (28.2%) were *MGMT* promoter-methylated, 6 (15.4%) were *MGMT* promoter-unmethylated, and 22 (56.4%) had missing *MGMT* promoter methylation data.

**Table 1.** Baseline characteristics of the patients in the FAS

Characteristics	Vebreltinib group (*n* = 42)	Control group (*n* = 39)	All (*n* = 81)
Age, years, median (IQR)	41.0 (35.0–48.0)	40.0 (32.0–49.0)	41.0 (35.0–49.0)
Male, *n* (%)	22 (52.4)	22 (56.4)	44 (54.3)
KPS score, median (IQR)	70 (60–80)	70 (60–80)	70 (60–80)
60–79, *n* (%)	29 (69.0)	27 (69.2)	56 (69.1)
≥80, *n* (%)	13 (31.0)	12 (30.8)	25 (30.9)
Baseline lesion diameter, *n* (%)			
≤3.0 cm	8 (19.0)	9 (23.1)	17 (21.0)
>3.0 cm	31 (73.8)	26 (66.7)	57 (70.4)
Missing	3 (7.1)	4 (10.3)	7 (8.6)
Time since initial diagnosis, months, median (IQR)	35.5 (20.6–67.0)	51.1 (25.0–65.4)	43.9 (20.6–67.0)
Histopathological diagnosis, *n* (%)			
Astrocytoma, *IDH*-mutant, grade 4	39 (92.9)	34 (87.2)	73 (90.1)
Glioblastoma, *IDH* wild-type	3 (7.1)	5 (12.8)	8 (9.9)
*ZM* fusion positive, *n* (%)	42 (100.0)	39 (100.0)	81 (100.0)
Tumor location, *n* (%)			
Frontal lobe	12 (28.6)	15 (38.5)	27 (33.3)
Temporal lobe	2 (4.8)	4 (10.3)	6 (7.4)
Parietal lobe	1 (2.4)	0 (0.0)	1 (1.2)
Occipital lobe	0 (0.0)	1 (2.6)	1 (1.2)
Insular lobe	3 (7.1)	0 (0.0)	3 (3.7)
Multiple lobes	24 (57.1)	19 (48.7)	43 (53.1)
*MGMT* promoter methylation status			
Positive	8 (19.0)	11 (28.2)	19 (23.5)
Negative	11 (26.2)	6 (15.4)	17 (21.0)
Missing	23 (54.8)	22 (56.4)	45 (55.6)
Previous antitumor treatments, *n* (%)			
Surgery	42 (100.0)	39 (100.0)	81 (100.0)
Gross total resection	27 (64.3)	23 (59.0)	50 (61.7)
Subtotal resection	13 (31.0)	12 (30.8)	25 (30.9)
Partial resection	2 (4.8)	4 (10.3)	6 (7.4)
Radiotherapy	42 (100.0)	39 (100.0)	81 (100.0)
Chemotherapy	42 (100.0)	39 (100.0)	81 (100.0)
Bevacizumab	7 (16.7)	5 (12.8)	12 (14.8)

IQR, interquartile range; KPS, Karnofsky performance status; IDH, isocitrate dehydrogenase; ZM, PTPRZ1-MET; MGMT, O^6^-methylguanine-DNA methyltransferase.

Among those in the vebreltinib group, 6 patients (14.3%) underwent surgical resection following discontinuation of study treatment, 1 (2.4%) received additional radiotherapy, and 9 (21.4%) received chemotherapy. In the control group, 1 patient (2.6%) underwent surgery, none received further radiotherapy, and 7 (17.9%) received additional chemotherapy (Table S1).

### Primary end point

**Fig. 2.**
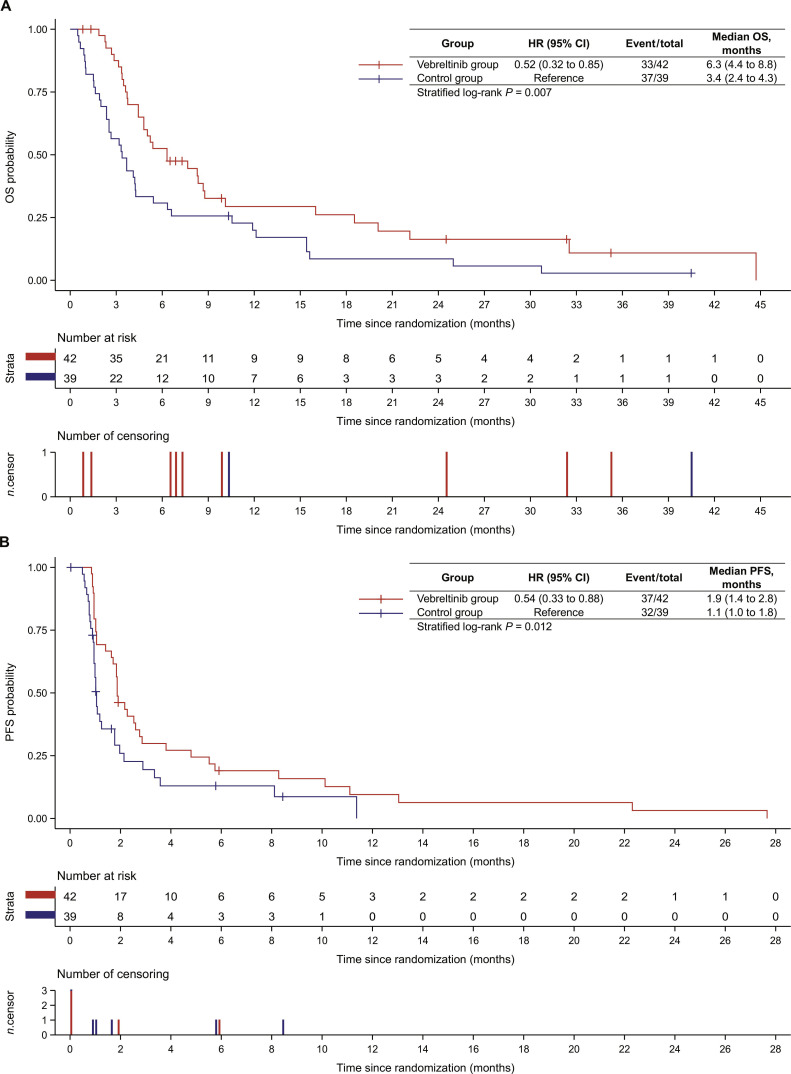
Kaplan–Meier curves in the full analysis set (*n* = 81). (A) Overall survival (OS). (B) Progression-free survival (PFS). HR, hazard ratio; CI, confidence interval; *n*.censor, number of censoring.

As of 2023 April 1, the median duration of follow-up was 5.9 (range, 0.8 to 44.7) months in the vebreltinib group and 3.4 (range, 0.5 to 40.5) months in the control group. In the FAS, a total of 33 deaths (78.6%) occurred in the vebreltinib group, compared with 37 deaths (94.9%) in the control group. Median OS was significantly longer among patients receiving vebreltinib (6.3 [95% CI, 4.4 to 8.8] months) than among those in the control group (3.4 [95% CI, 2.4 to 4.3] months), with an HR of 0.52 (95% CI, 0.32 to 0.85; stratified log-rank *P* = 0.007; Fig. 2A). At 6 months, the estimated OS rate was 53% (95% CI, 36% to 67%) in the vebreltinib group and 31% (95% CI, 17% to 45%) in the control group; at 12 months, the rates were 29% (95% CI, 16% to 45%) and 20% (95% CI, 9% to 34%), respectively. In the ITT population, the median OS was 6.3 (95% CI, 4.4 to 8.7) months in the vebreltinib group and 3.7 (95% CI, 2.4 to 5.4) months in the control group (Fig. S1A), with an HR of 0.60 (95% CI, 0.37 to 0.97; stratified log-rank *P* = 0.034), further supporting the OS benefit associated with vebreltinib treatment.

### Secondary end points

PFS was also improved in the vebreltinib group. Among patients in the FAS, 33 of 42 patients in the vebreltinib group and 26 of 39 in the control group experienced disease progression, while 4 and 6 patients, respectively, died without documented progression. The median PFS was 1.9 (95% CI, 1.4 to 2.8) months with vebreltinib, compared with 1.1 (95% CI, 1.0 to 1.8) months with control treatment (HR, 0.54; 95% CI, 0.33 to 0.88; stratified log-rank *P* = 0.012; Fig. 2B). The ITT population showed consistent PFS results, favoring vebreltinib (median PFS of 1.9 [95% CI, 1.4 to 2.8] months) versus the control group (median PFS of 1.1 [95% CI, 1.0 to 1.8] months; HR, 0.61; 95% CI, 0.37 to 1.01; stratified log-rank *P* = 0.047; Fig. S1B).

Among 42 patients in the vebreltinib group, 1 complete response (2.4%) and 3 partial responses (7.1%) were observed, resulting in an ORR of 9.5% (95% CI, 2.7% to 22.6%) in the vebreltinib group. In the control group, 1 of 39 patients (2.6%; 95% CI, 0.1% to 13.5%) achieved a complete response, with no partial responses reported. ORR was comparable between groups (*P* = 0.361; Table S2). Patient-reported outcomes and performance status assessments demonstrated generally comparable trends between the 2 groups over time. Improvements were observed in KPS scores, global health status (summary score), and emotional functioning as measured by the EORTC QLQ-C30. Symptom domains related to seizure severity and visual disturbances, as assessed by the QLQ-BN20, also showed similar temporal patterns in both treatment groups (Figs. S2 and S3).

### Subgroup analysis of the efficacy end points

**Fig. 3.**
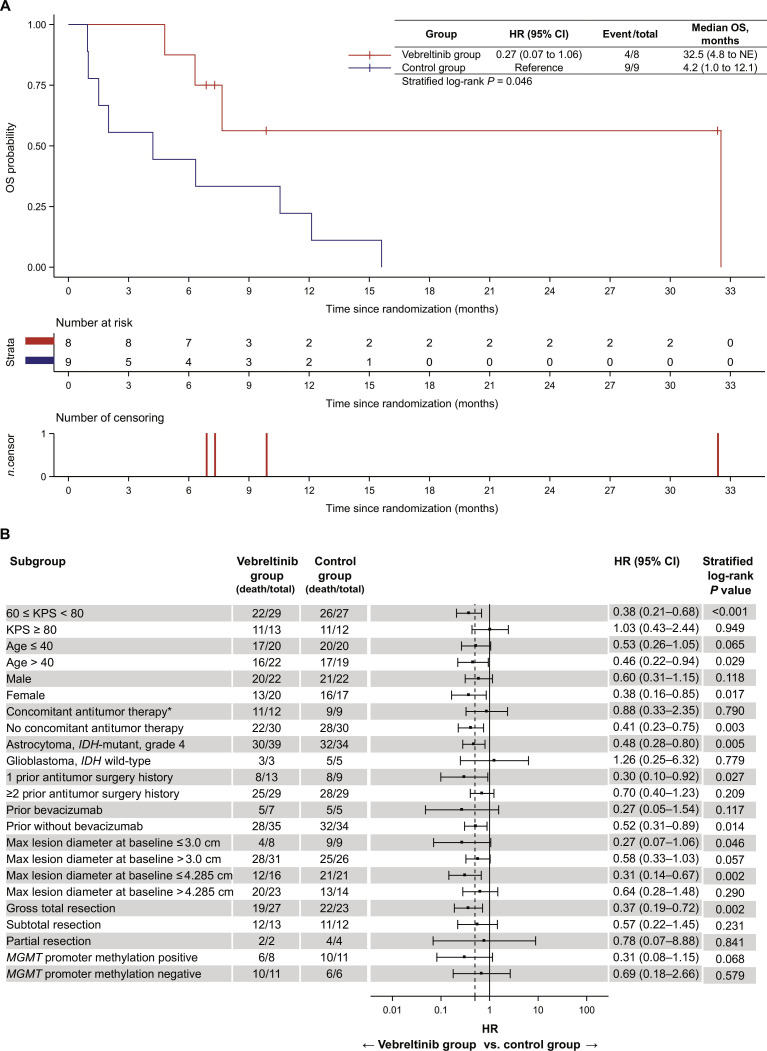
Subgroup analysis of overall survival. (A) Kaplan–Meier estimates of OS in patients with a maximum lesion diameter at baseline of ≤3.0 cm. (B) Forest plot of OS across subgroups. HR, hazard ratio; CI, confidence interval; NE, not estimatable; n.censor, number of censoring; KPS, Karnofsky Performance Status; IDH, isocitrate dehydrogenase; max, maximum; MGMT, O^6^-methylguanine-DNA methyltransferase. Asterisk (*) indicates that concomitant antitumor therapy refers to antitumor treatment with nonstudy drugs administered during the survival follow-up period. Note that 4.285 cm was the median baseline value of maximum lesion diameter.

In subgroup analyses stratified by baseline tumor size, patients with a maximum lesion diameter of ≤3.0 cm experienced prolonged median OS in the vebreltinib group (32.5 [95% CI, 4.8 to not estimable {NE}] months) compared with the control group (4.2 [95% CI, 1.0 to 12.1] months), with an HR of 0.27 (95% CI, 0.07 to 1.06; stratified log-rank *P* = 0.046; Fig. 3A). Among patients with tumors >3.0 cm, median OS was 4.8 (95% CI, 3.5 to 8.3) months in the vebreltinib group compared with 2.9 (95% CI, 1.9 to 4.2) months in the control group. The HR of 0.58 (95% CI, 0.33 to 1.03; stratified log-rank *P* = 0.057) indicated a favorable trend toward improved survival with vebreltinib, although the difference did not reach statistical significance (Fig. S4).

Forest plot analysis showed that vebreltinib was associated with a reduced risk of death across most predefined subgroups (Fig. 3B). Notable risk reductions in mortality (>50%) were observed among patients with KPS scores of ≥60 and <80, those aged >40 years, female patients, individuals without concomitant antitumor therapy, patients with astrocytoma, *IDH*-mutant, WHO grade 4, those with a history of one prior surgery, prior bevacizumab use, and patients with a maximum lesion diameter at baseline of ≤3.0 cm or ≤4.285 cm (the median baseline value of maximum lesion diameter). In the *IDH*-mutant subgroup, vebreltinib treatment resulted in a median OS of 7.7 (95% CI, 4.44 to 10.12) months compared to 3.3 (95% CI, 2.00 to 4.21) months in the control group (HR, 0.48; 95% CI, 0.28 to 0.80; stratified log-rank *P* = 0.005). In the *IDH* wild-type subgroup, median OS was 5.0 (95% CI, 2.27 to NE) months and 5.4 (95% CI, 1.64 to NE) months, respectively (HR, 1.26; 95% CI, 0.25 to 6.32; stratified log-rank *P* = 0.779). In a post hoc subgroup analysis stratified by extent of surgical resection, survival was improved with vebreltinib in patients who had undergone gross total resection (8.3 months versus 3.3 months; HR, 0.37; 95% CI, 0.19 to 0.72; stratified log-rank *P* = 0.002). In exploratory analyses stratified by *MGMT* promoter methylation status, median OS favored the vebreltinib group in both subgroups. Among patients with *MGMT*-methylated tumors, median OS was 8.3 (95% CI, 2.86 to NE) months in the vebreltinib group and 3.4 (95% CI, 1.54 to 10.55) months in the control group (HR, 0.31; 95% CI, 0.08 to 1.15; stratified log-rank *P* = 0.068). Among patients with *MGMT*-unmethylated tumors, median OS was 4.8 (95% CI, 2.27 to 8.67) months with vebreltinib and 2.5 (95% CI, 0.56 to NE) months in the control group (HR, 0.69; 95% CI, 0.18 to 2.66; stratified log-rank *P* = 0.579).

Subgroup analyses of PFS revealed a consistent pattern. In patients with tumors ≤ 3.0 cm, median PFS was longer with vebreltinib than with control (5.3 months versus 1.2 months; HR, 0.33; 95% CI, 0.10 to 1.09; stratified log-rank *P* = 0.059), although this difference did not reach statistical significance (Fig. S5). Similarly, among patients with tumors >3.0 cm, vebreltinib was associated with a significantly longer median PFS compared with control (1.8 months versus 1.0 month; HR, 0.47; 95% CI, 0.26 to 0.86; stratified log-rank *P* = 0.011; Fig. S6). The forest plot of PFS indicated that most subgroups favored vebreltinib over control treatment (Fig. S7). The median PFS in the *IDH*-mutant subgroup was 1.89 (95% CI, 1.41 to 2.86) months with vebreltinib versus 1.02 (95% CI, 0.92 to 1.77) months in the control group (HR, 0.54; 95% CI, 0.31 to 0.92; stratified log-rank *P* = 0.019). In the *IDH* wild-type subgroup, median PFS was 1.9 (95% CI, 0.95 to NE) months with vebreltinib and 1.1 (95% CI, 0.59 to NE) months in the control group (HR, 0.46; 95% CI, 0.08 to 2.57; stratified log-rank *P* = 0.349).

### Safety

The median treatment exposure duration was 102.0 (range, 19.0 to 1,071.0) d in the vebreltinib group. In the control group, the median exposure was 47.0 (range, 4.0 to 388.0) d for patients receiving temozolomide and 175.0 (range, 175.0 to 175.0) d for the single patient treated with cisplatin plus etoposide.

AEs were reported in 42 of 43 patients (97.7%) receiving vebreltinib and in 35 of 41 patients (85.4%) in the control group (Table 2). Grade ≥3 AEs occurred in 22 patients (51.2%) in the vebreltinib group and 21 patients (51.2%) in the control group. The most frequently reported AEs in the vebreltinib group were epilepsy seizure (32.6%) and vomiting (32.6%), whereas vomiting (34.1%) and constipation (26.8%) were the most common in the control group (Tables S3 and S4). AEs leading to death occurred in 3 patients (7.0%) in the vebreltinib group and 2 patients (4.9%) in the control group; none were considered related to the study treatment. Treatment discontinuation due to AEs occurred in 3 patients (7.0%) in the vebreltinib group, while no patients in the control group permanently discontinued treatment because of AEs.

**Table 2.** Summary of AEs in the safety analysis set

Events, *n* (%)	Vebreltinib group (*n* = 43)	Control group (*n* = 41)
Any AE	42 (97.7)	35 (85.4)
Any TRAE	26 (60.5)	20 (48.8)
SAE	18 (41.9)	13 (31.7)
Treatment-related SAE	0 (0.0)	1 (2.4)
Any AE leading to death	3 (7.0)	2 (4.9)
TRAE leading to death	0 (0.0)	0 (0.0)
Grade ≥3 AE	22 (51.2)	21 (51.2)
Grade ≥3 TRAE	3 (7.0)	5 (12.2)
AE leading to study withdrawal	0 (0.0)	0 (0.0)
TRAE leading to study withdrawal	0 (0.0)	0 (0.0)
AE leading to temporary discontinuation of study drug	12 (27.9)	7 (17.1)
TRAE leading to temporary discontinuation of study drug	2 (4.7)	2 (4.9)
AE leading to dose adjustment of study drug	3 (7.0)	2 (4.9)
TRAE leading to dose adjustment of study drug	0 (0.0)	2 (4.9)
AE leading to permanent discontinuation of study drug	3 (7.0)	0 (0.0)
TRAE leading to permanent discontinuation of study drug	0 (0.0)	0 (0.0)

AE, adverse event; TRAE, treatment-related adverse event; SAE, serious adverse event.

Treatment-related AEs (TRAEs) were observed in 26 of 43 patients (60.5%) in the vebreltinib group and 20 of 41 patients (48.8%) in the control group. The most common TRAEs in the vebreltinib group included rash (25.6%) and peripheral edema (14.0%), while vomiting (29.3%) and nausea (17.1%) were most frequent in the control group (Tables S5 and S6). SAEs occurred in 18 patients (41.9%) in the vebreltinib group and in 13 patients (31.7%) in the control group. No treatment-related SAEs were reported in the vebreltinib group, whereas one treatment-related SAE (2.4%) occurred in the control group. The most common SAEs in the vebreltinib group included epilepsy seizure (9.3%) and headache (9.3%), while in the control group, the most frequent SAEs were headache (12.2%) and epilepsy seizure (7.3%) (Tables S5 and S6).

## Discussion

In this multicenter, randomized trial, we investigated the efficacy and safety of vebreltinib, a selective *MET* inhibitor, in patients with previously treated astrocytoma, *IDH*-mutant, grade 4, or glioblastoma, *IDH* wild-type, harboring the *ZM* fusion gene. The study met its primary end point, demonstrating that vebreltinib significantly prolonged OS compared with standard chemotherapy. The median OS was 6.3 months in the vebreltinib group, as compared with 3.4 months in the control group, corresponding to a 48% reduction in the risk of death. PFS was also improved, with a median of 1.9 months in the vebreltinib group versus 1.1 months in the control group. Objective responses were more frequent in the vebreltinib arm (9.5% versus 2.6%), and patient-reported outcomes indicated better preservation of quality of life. The safety profile of vebreltinib was manageable, with no treatment-related deaths or SAEs observed. The prolonged survival was consistently observed in patients with astrocytoma, *IDH*-mutant, grade 4, who comprised the majority of the study population. However, given the limited sample size in the glioblastoma, the outcomes in the *IDH* wild-type subgroup remain inconclusive and warrant further investigation.

Although *MET* has long been recognized as a potential therapeutic target in glioblastoma, most clinical investigations of *MET* inhibitors have demonstrated limited efficacy [20,21]. In a phase Ib/II trial of capmatinib in patients with recurrent glioblastoma harboring *MET* amplification and *PTEN* alterations, no objective responses were observed; the best response was stable disease in 30% of patients, highlighting the limited clinical activity [22]. Similarly, in the Spanish Group for Research in Neuro-oncology (GEINO) 1402 trial, the addition of crizotinib to standard radiotherapy and temozolomide in newly diagnosed glioblastoma yielded a median OS of 22.6 months, but molecular biomarkers, including *MET* amplification, failed to correlate with treatment response, suggesting that efficacy may not be *MET* dependent [23]. Vebreltinib has high CNS penetration and enhanced kinase selectivity and has demonstrated promising activity. Preclinical studies showed that vebreltinib effectively inhibits phosphorylation of MET and downstream signal transducer and activator of transcription 3 (*STAT3*), providing a strong mechanistic rationale for its clinical evaluation [15]. In our multicenter, randomized trial, vebreltinib significantly prolonged OS among patients with astrocytoma, *IDH*-mutant, grade 4, and glioblastoma, *IDH* wild-type, harboring the *ZM* fusion. The median OS in the vebreltinib group was prolonged by approximately 3 months. This clinical benefit is particularly notable, given the aggressive nature of *ZM* fusion-positive gliomas. Survival analyses have suggested that patients with *ZM*-fused secondary glioblastoma have a median OS of approximately 4 months, significantly shorter than those without the fusion [9]. Previous studies have also shown that the *ZM* fusion promotes *MET* overexpression, persistent activation of downstream signaling, and recruitment of tumor-associated macrophages, thereby contributing to tumor progression and immune evasion [15]. Therefore, vebreltinib’s ability to counteract these oncogenic processes underscores its potential as a biomarker-driven therapeutic option for this glioma subtype.

Currently, the standard of care for glioblastoma is nontargeted and consists of maximal safe surgical resection, followed by radiotherapy with concomitant and adjuvant temozolomide. While this multimodal approach provides modest survival benefits, therapeutic outcomes remain unsatisfactory, underscoring the need for molecularly targeted interventions. Targeted therapeutic approaches are urgently needed for glioblastoma with *ZM* fusion. In a pilot exploration of crizotinib in an 8-year-old male patient with recurrent glioblastoma harboring the *ZM* fusion, crizotinib led to a partial response of the primary lesion; however, new lesions were also observed, followed by rapid progression and death [11]. As a first-generation *MET* inhibitor, cabozantinib has shown limited efficacy, possibly due to its poor blood–brain barrier penetration and low kinase selectivity. Another oral *MET* tyrosine kinase inhibitor, tepotinib, has shown preliminary activity in a case of disseminated glioblastoma, *IDH* wild-type, with *ZM* fusion [24]. Nonetheless, evidence supporting the efficacy of *MET* inhibitors remains limited in larger populations. Vebreltinib, as evaluated in the present trial, provides valuable phase III data supporting *MET*-targeted therapy for patients with *IDH*-mutant high-grade gliomas harboring the *ZM* fusion. Although this study was initiated before the adoption of the WHO CNS5 classification and enrolled a small number of patients with glioblastoma, *IDH* wild-type, the results nonetheless demonstrate a therapeutic effect. The survival improvement achieved with vebreltinib appears favorable, given that prior Food-and-Drug-Administration-approved therapies for glioblastoma have conferred median OS gains of only 1.8 to 2.5 months [25–27]. Herein, we also observed an improvement in PFS (median, 1.9 months versus 1.1 months; HR, 0.54; 95% CI, 0.33 to 0.88; *P* = 0.012). However, given the relatively short PFS (less than 2 months), these results may be subject to bias arising from the 1-month interval between follow-up assessments in the early phase of the study.

Effective CNS penetration is a key requirement for targeted therapies in gliomas. Vebreltinib was designed to optimize brain exposure through its physicochemical and pharmacokinetic properties. Structurally, vebreltinib exhibits relatively high lipophilicity, which facilitates passive diffusion across the blood–brain barrier; the presence of difluoromethyl and cyclopropyl moieties further contributes to this lipophilic profile [28]. In addition, vebreltinib is not a substrate of P-glycoprotein, a major efflux transporter expressed on the luminal surface of brain endothelial cells, thereby reducing active drug extrusion from the CNS and supporting sustained intracranial drug exposure [29]. Preclinical studies using intracranial xenograft models harboring the *ZM* fusion demonstrated higher baseline activation of *MET* and downstream *STAT3* signaling compared with fusion-negative tumors. Treatment with vebreltinib resulted in marked suppression of *MET* and *STAT3* phosphorylation, reduced tumor cell proliferation, and decreased angiogenesis, providing functional evidence of effective target engagement within the CNS [15]. Consistent with these findings, pharmacokinetic data from a phase I clinical study showed that vebreltinib concentrations in cerebrospinal fluid collected on day 15 reached approximately 3% to 8% of corresponding plasma levels and increased with dose, supporting its ability to penetrate the CNS [15]. Collectively, these data provide a mechanistic and translational rationale for the observed clinical activity of vebreltinib in patients with *ZM* fusion-positive gliomas.

Notably, subgroup analyses suggested a pronounced OS benefit among patients with baseline lesions of ≤3.0 cm in maximum diameter, with patients in the vebreltinib group exhibiting a median OS of 32.5 months. While these findings are encouraging, they must be interpreted with caution due to the limited sample size. Two patients demonstrated exceptionally long survival, suggesting a possible signal that early intervention in patients with a low tumor burden may yield substantial benefit. These results, although exploratory, warrant further investigation and suggest that tumor size may serve as a predictive marker for response to *MET*-targeted therapy.

A patient underwent right frontal lobe tumor resection in 2020 October, with pathological results confirming astrocytoma, WHO grade 4, *IDH*-mutant. From 2020 November to 2021 January, the patient received concurrent chemoradiotherapy with temozolomide, followed by 6 cycles of adjuvant temozolomide from 2021 January to June. An MRI reexamination in 2021 June revealed tumor progression, after which the patient was enrolled in this trial and randomized to the vebreltinib group, achieving a partial response as the best overall response. On 2022 March 2, MRI indicated progressive disease, with a PFS of 8.3 months. Subsequent treatments included surgery (2022 March), followed by chemotherapy, a vascular endothelial growth factor receptor inhibitor, and an anti-programmed cell death protein 1 antibody (tislelizumab). The patient ultimately died in 2022 October. This case exemplifies the potential clinical benefit of vebreltinib in patients achieving an objective response, as reflected by prolonged PFS and extended OS relative to the median outcomes of the broader cohort.

Notably, vebreltinib exhibited a favorable safety profile, characterized predominantly by grade 1 to 2 AEs. The incidence of grade ≥3 AEs was comparable between the vebreltinib and control groups (51.2% versus 51.2%), and no unexpected toxicities emerged. SAEs occurred, yet none were attributed to the study drug. The rates of AEs leading to death were similar between the vebreltinib and control groups (7.0% versus 4.9%), and these events were mainly related to the primary disease rather than drug toxicity. Common AEs observed with vebreltinib, such as rash and peripheral edema, were primarily low grade and manageable. These findings underscore the tolerability of vebreltinib and support its potential use in combination with standard care regimens.

Despite the promising results, this study has several limitations. The rarity of the *ZM* fusion in patients with *IDH*-mutant high-grade gliomas presents challenges in patient accrual and limits the generalizability of our findings. Moreover, as a multicenter, open-label trial, the study design inherently carries the potential for bias in outcome assessment. Although the observed survival benefit and safety profile of vebreltinib are encouraging, validation in larger, randomized, and ideally blinded trials is necessary. In addition, given the molecular complexity and intratumoral heterogeneity of gliomas, future research should prioritize elucidating mechanisms of resistance to *MET* inhibition. Combination strategies, such as pairing *MET* inhibitors with agents targeting parallel oncogenic pathways or modulating the tumor microenvironment, may further enhance therapeutic efficacy. Another limitation of this study is the inability to definitively differentiate between recurrence and pseudoprogression in the absence of histopathological confirmation. While pseudoprogression typically occurs within the first 3 months following radiotherapy [30], our study included patients who had been previously treated for high-grade gliomas, with a median time of over 40 months since the initial diagnosis. Thus, the likelihood of pseudoprogression is reduced. However, any potential bias from pseudoprogression would have similarly affected both treatment groups and is unlikely to have systematically influenced the observed survival outcomes. Besides, *MGMT* promoter methylation status was missing in more than half of the study population, limiting the ability to perform robust subgroup analyses and to fully assess its predictive or prognostic role, particularly in patients receiving temozolomide rechallenge. An important limitation of this study relates to the inclusion of both astrocytoma, *IDH*-mutant, WHO grade 4, and glioblastoma, *IDH* wild-type, within the same analysis cohort. These entities are now recognized as biologically and clinically distinct diseases under the WHO 2021 classification [31]. However, this trial was initiated prior to the adoption of this classification, and the primary analyses were therefore conducted according to the original protocol-defined population.

In conclusion, the FUGEN trial showed that vebreltinib was associated with improved OS in patients with previously treated high-grade glioma harboring the *ZM* fusion, specifically in the subgroup of patients with astrocytoma, *IDH*-mutant, WHO grade 4 disease. The treatment was generally well tolerated, with no unexpected safety signals or treatment-related deaths. Collectively, these findings suggest that vebreltinib may represent an important therapeutic option for patients with *ZM* fusion gliomas.

## Ethical Approval

The trial was conducted in accordance with the Declaration of Helsinki and applicable clinical practice guidelines. Ethical approval was secured from the institutional review board or independent ethics committee at each participating site (see the Supplementary Materials). Written informed consent was obtained from all patients or their legal representatives.

## Data Availability

The authors confirm that the data supporting the findings of this study are available within the article and its Supplementary Materials. Other study data may be shared upon submission of a request to Beijing Pearl Biotechnology Co. Ltd. The data request will be reviewed, and, if agreed, the requestors will need to sign a data sharing agreement.
