# Supplementary material for: Vebreltinib for Previously Treated Astrocytoma, IDH-Mutant, Grade 4, and Glioblastoma, IDH Wild-Type with PTPRZ1–MET Fusion Gene: A Multicenter, Phase III Randomized, Open-Label Trial
Source: Cancer Commun (Lond). 2026 Mar 11;46:0019. doi: 10.34133/cancomm.0019 (PMC12976379; doi:10.34133/cancomm.0019)
Supplement: Supplementary 1 — Tables S1 to S6 Figs. S1 to S7 [file cancomm.0019.f1.zip › CANCOMM-D-25-00184-Supplementary-Final.docx]

**Vebreltinib for** **previously treated astrocytoma, *IDH*-mutant, grade 4 and** **glioblastoma, *IDH* wild-type with *PTPRZ1-MET* fusion gene: a multicenter, phase III randomized, open-label trial**

Running title: Vebreltinib for high-grade gliomas

Zhaoshi Bao^1,2†^, Yake Xue^3†^, Yanhui Liu^4†^, Shouwei Li^5†^, Liang Wang^6†^, Yan Qu^6^, Yonggao Mou^7^, Rutong Yu^8^, Jinsong Wu^9^, Yu Yao^9^, Kai Shu^10^, Guangyuan Hu^10^, Linbo Cai^11^, Wenbin Li^12^, Xiaoguang Qiu^13^, Yunqian Li^14^, Lei Zhang^15^, Songtao Qi^16^, Ying Ji^17^, Chunxiao Ma^18^, Wenbin Ma^19^, Gang Li^20^, Rongjie Tao^21^, Chongran Sun^22^, Ligang Chen^23^, Sheng-Qing Lv^24^, Peng Liang^25^, Hao Pan^26,27^, Woo Yat Ming Peter^28^, Chan Tat Ming Danny^28^, Qing Mao^4*^, Xinting Wei^3*^, Tao Jiang,^1,2*^ On behalf of the FUGEN investigators

^1^ Beijing Neurosurgical Institute, Beijing, P. R. China

^2^ Department of Neurosurgery, Beijing Tiantan Hospital, Capital Medical University, Beijing, P. R. China

^3^ Department of Neurosurgery, The First Affiliated Hospital of Zhengzhou University, Zhengzhou, Henan, P. R. China

^4^ Department of Neurosurgery, West China Hospital, Sichuan University, Chengdu, Sichuan, P. R. China

^5^ Department of Neurosurgery, Sanbo Brain Hospital, Capital Medical University, Beijing, P. R. China

^6^ Department of Neurosurgery, Tangdu Hospital, Fourth Military Medical University, Xi’an, Shanxi, P. R. China

^7^ Department of Neurosurgery/Neuro-oncology, State Key Laboratory of Oncology in South China, Guangdong Provincial Clinical Research Center for Cancer, Sun Yat-sen University Cancer Center, Guangzhou, Guangdong, P. R. China

^8^ Department of Neurosurgery, Affiliated Hospital of Xuzhou Medical University, Xuzhou, Jiangsu, P. R. China

^9^ Department of Neurosurgery, Huashan Hospital, Fudan University, Shanghai, P. R. China

^10^ Department of Neurosurgery, Tongji Hospital, Tongji Medical College of Huazhong University of Science and Technology, Wuhan, Hubei, P. R. China

^11^ Department of Neurosurgery, Guangdong Sanjiu Brain Hospital, Guangzhou, Guangdong, P. R. China

^12^ Department of Neuro-oncology, Beijing Tiantan Hospital, Capital Medical University, Beijing, P. R. China

^13^ Department of Radiation Oncology, Beijing Tiantan Hospital, Capital Medical University, Beijing, P. R. China

^14^ Department of Neurosurgery, The First Hospital of Jilin University, Changchun, Jilin, P. R. China

^15^ Department of Neurosurgery, Shengjing Hospital of China Medical University, Shenyang, Liaoning, P. R. China

^16^ Department of Neurosurgery, Nanfang Hospital, Southern Medical University, Guangzhou, Guangdong, P. R. China

^17^ Department of Neurosurgery, The First Affiliated Hospital of University of Science and Technology of China, Division of Life Sciences and Medicine, University of Science and Technology of China, Hefei, Anhui, P. R. China

^18^ Department of Neurosurgery, Henan Provincial People's Hospital, People's Hospital of Zhengzhou University, Zhengzhou, Henan, P. R. China

^19^ Department of Neurosurgery, Peking Union Medical College Hospital, Beijing, P. R. China

^20^ Department of Neurosurgery, Qilu Hospital of Shandong University, Jinan, Shandong, P. R. China

^21^ Department of Neurosurgery, Shandong Tumor Hospital, Jinan, Shandong, P. R. China

^22^ Department of Neurosurgery, The Second Affiliated Hospital, Zhejiang University School of Medicine, Hangzhou, Zhejiang, P. R. China

^23^ Department of Neurosurgery, The Affiliated Hospital of Southwest Medical University, Luzhou, Sichuan, P. R. China

^24^ Department of Neurosurgery, Xinqiao Hospital, Third Military Medical University (Army Medical University), Chongqing, P. R. China

^25^ Department of Neurosurgery, Harbin Medical University Cancer Hospital, Harbin, Heilongjiang, P. R. China

^26^ Department of Neurosurgery, General Hospital of Eastern Theater Command of Chinese People's Liberation Army, Nanjing, Jiangsu, P. R. China

^27^ Department of Neurosurgery, The Affiliated BenQ Hospital of Nanjing Medical University, Nanjing, Jiangsu, P. R. China

^28^ The Otto Wong Brain Tumour Centre, Division of Neurosurgery, Department of Surgery, The Chinese University of Hong Kong, New Territories, Hong Kong, P. R. China

**^*^Corresponding authors:**

Tao Jiang, Beijing Neurosurgical Institute, 119 Nanxihuan Xi Lu, Fengtai District, Beijing 100070, P. R. China; Department of Neurosurgery, Beijing Tiantan Hospital, Capital Medical University, 119 Nanxihuan Xi Lu, Fengtai District, Beijing 100070, P. R. China; Email: taojiang1964@163.com

Xinting Wei, Department of Neurosurgery, The First Affiliated Hospital of Zhengzhou University, No.1 Jianshe Dong Road, Erqi District, Zhengzhou 450052, Henan, P. R. China; Email: fccweixt@zzu.edu.cn

Qing Mao, Department of Neurosurgery, West China Hospital, Sichuan University, No.37 Guoxue Alley, Wuhou District, Chengdu 610041, Sichuan, P. R. China; Email: qingmao2000@163.com

^†^These authors contributed equally to this work.

**Supplementary Materials**

**Inclusion criteria**

1. Male or female with 18 ≤ age ≤ 65 years;
2. Histologically confirmed secondary glioblastoma (progression from lower-grade glioma to glioblastoma) or isocitrate dehydrogenase (*IDH*)-mutant glioblastoma (Histology and *IDH* test reports from other hospitals are acceptable);

Note: The inclusion criteria listed here are consistent with the original protocol. Diagnostic terms in the original protocol are based on the 2016 World Health Organization (WHO) classification [1]. Contemporary diagnostic terms adhering to the 2021 WHO criteria [2] are applied elsewhere in this study for clinical context. This does not alter patient eligibility.

1. The latest surgical sample was confirmed to be positive for the *PTPRZ1-MET* *(ZM)* fusion gene through molecular pathological testing in the central laboratory;
2. Those who had previously received radiotherapy (including gamma knife, cyberknife, etc.) and temozolomide treatment and had tumor recurrence, or who had received temozolomide but were not suitable for radiotherapy, or who had received radiotherapy but were intolerant to temozolomide (absolute neutrophil count <0.5 × 10⁹/L or platelet count <10 × 10⁹/L or grade 3-4 non-hematologic toxicity excluding hair loss, nausea, vomiting, etc.);
3. Did not receive glucocorticoid treatment within 5 days prior to enrollment, or received stable or reduced doses of glucocorticoid treatment within 5 days prior to enrollment;
4. Pre-enrollment laboratory test results are consistent with:
   (1) Blood count: platelet count ≥75 × 10⁹/L; absolute neutrophil count ≥1.5 × 10⁹/L; hemoglobin >90 g/L;
   (2) Blood biochemistry: aspartate aminotransferase (AST) ≤ 3 × upper limit of normal (ULN); alanine aminotransferase (ALT) ≤ 3 × ULN; total bilirubin ≤ 2 × ULN; serum creatinine ≤ 1.5 × ULN; urea nitrogen ≤ 1.5 × ULN; serum amylase ≤ 1.5 × ULN or 1.5 × ULN < serum amylase ≤ 2 × ULN without evidence of pancreatic disease; serum lipase ≤ 1.5 × ULN or 1.5 × ULN < serum lipase ≤ 2 × ULN without evidence of pancreatic disease; fasting serum triglyceride level ≤ 2.5 × ULN;
   (3) Coagulation function: prothrombin time international standardized ratio (INR) ≤2.0;
5. Karnofsky Performance Status (KPS) ≥60, be able to swallow the drug and keep it orally;
6. Life expectancy of ≥3 months;
7. Female participants must have a negative serum beta-human chorionic gonadotropin pregnancy test within 7 days prior to enrollment if of childbearing potential, and are required to use adequate contraception (i.e., intrauterine device, spermicidal barrier, condom, hormonal contraceptives, or abstinence) during their participation in the study and for 3 months following the last dose administration; and
8. Voluntarily participate in this study and sign the informed consent form, and be able to understand and comply with the requirements of the study.

**Exclusion criteria**

1. Previously received cellular-mesenchymal epithelial transition factor (c-MET) inhibitors or hepatocyte growth factor (HGF)-targeted drugs;
2. Antibody oncology drugs received within 30 days prior to study enrollment;
3. Previously received carmustine extended-release implants or intralesional radiotherapy;
4. Patients who could not have brain magnetic resonance imaging (MRI);
5. Active bleeding detected by transcranial computed tomography or MRI scan before enrollment;
6. Uncompensated hypertension with systolic blood pressure >150 mmHg and/or diastolic blood pressure >100 mmHg after treatment with antihypertension drugs;
7. Decompensated heart failure (New York Heart Association classes III and IV), unstable angina, acute myocardial infarction, persistent and clinically significant arrhythmias within 3 months prior to enrollment;
8. Severe trauma or infection affecting current antitumor therapy within 4 weeks prior to enrollment;
9. Grade 3 or higher chronic toxic reactions (excluding hair loss) according to the National Cancer Institute Common Adverse Event Evaluation Criteria version 5.0 (NCI-CTCAE 5.0);
10. Major surgery (excluding glioma surgery) performed within 4 weeks prior to enrollment; individuals who have undergone bone marrow biopsy, open biopsy, or intracranial biopsy within 7 days prior to screening;
11. Anti-human immunodeficiency virus (HIV) (+), or both anti-hepatitis C virus (HCV) and HCV-RNA (+), or hepatitis B surface antigen (HBsAg) (+) and hepatitis B virus (HBV) DNA >1000 IU/ml. If HBsAg (+) but HBV-DNA level between 1000 ~ 10,000 IU/ml, and patients were willing to use anti-viral therapy during the study period, they can be enrolled;
12. Patients with other malignancies within the past 5 years were excluded, except for carcinoma in situ of the cervix, squamous cell carcinoma of the skin, or localized basal cell skin cancer;
13. Long-term continuous use of hematopoietic growth factor (including granulocyte colony-stimulating factor, macrophage knockdown-stimulating factor, or interleukin-11) or platelet transfusion is required to maintain platelet count ≥75 × 10⁹/L and absolute neutrophil count ≥1.5 × 10⁹/L;
14. Pregnancy or breastfeeding, or plan to be pregnant during the study period;
15. Other study drugs used within 30 days prior to the first administration of the investigational drug;
16. Unsuitable to participate in this clinical trial, judged by the investigators.

**Ethical approval numbers of participating sites**

| Site Name | Ethical Approval Numbers |
| --- | --- |
| Beijing Tiantan Hospital, Capital Medical University | YW2018-015-02  YW2018-015-06  YW2018-015-10 |
| West China Hospital of Sichuan University | 2018 Clinical Trial (Chemical Drug) Approval (164) |
| Tangdu Hospilal, Fourth Military Medical University | 201811-01  K201905-05  202112-37 |
| The First Affiliated Hospital of Zhengzhou University | Drug-2018-149  Ammendment-2019-102 |
| Shandong Tumor Hospital | SDZLZC2019-042-01  SDZLZC2019-042-04 |
| Qilu Hospital of Shandong University | 2018071  2018071 (1) |
| The First Affiliated Hospital with Nanjing Medical University (Jiangsu Province Hospital) | 2018-MD-300  2018-MD-300. A1  2018-MD-300. A2 |
| Affiliated Hospital of Xuzhou Medical University | XYFY2018-YL104-02  XYFY2018-YL104-05  XYFY2018-YL104-16 |
| Tianjin Medical University General Hospital | IRB2018-122-01  IRB2018-122-02 |
| Huashan Hospital Fudan University | (2019) Clinical Review No. (304)  (2019) Clinical Review No. (304) Ammendment 1  (2019) Clinical Review No. (304) Ammendment 2 |
| The Second Affiliated Hospital, Zhejiang University School of Medicine | (2018) Clinical Review Drug No. (738)  (2019) Clinical Review Drug No. (289)  (2021) Clinical Review Drug No. (670) |
| Peking Union Medical College Hospital | KS2018471  KS2019234  KS2021634 |
| Sanbo Brain Hospital, Capital Medical University | SBNK-2018-042-01  SBNK-2018-042-02  SBNK-2018-042-06 |
| The First Hospital of Jilin University | 19Y021-001  19Y021-002  19Y021-007 |
| Shengjing Hospital of China Medical University | 2018PS95  2018PS95 (X1)  2018PS95 (X2) |
| Nanfang Hospital, Southern Medical University | NFEC-2019-006 |
| Guangdong Provincial Clinical Research Center for Cancer, Sun Yat-sen University Cancer Center | A2019-004-0l  A2019-004-02 |
| Tianjin Huanhu Hospital | (Jinhuan) Clinical Review No. (2019-62)  (Jinhuan) Clinical Review No. (2021-85) |
| Tongji Medical College of Huazhong University of Science and Technology | Clinical Review No. (121)  [2019] Clinical Review No. (121)-1  [2019] Clinical Review No. (121)-2 |

**Supplementary Table S1. Concomitant and subsequent anti-tumor treatments in the full analysis set**

| **Treatment type** | **Vebreltinib group (*n* = 42)** | **Control group (*n* = 39)** |
| --- | --- | --- |
| Concomitant treatments upon progression, *n* (%) | | |
| Any anti-tumor therapy* | 14 (33.3) | 4 (10.3) |
| Bevacizumab | 10 (23.8) | 3 (7.7) |
| Subsequent treatments after discontinuation of study treatment, *n* (%) | | |
| Received surgery, *n* (%) | 6 (14.3) | 1 (2.6) |
| Received radiotherapy, *n* (%) | 1 (2.4) | 0 (0.0) |
| Received chemotherapy, *n* (%) | 9 (21.4) | 7 (17.9) |
| Received other treatments, *n* (%) | 1 (2.4) | 2 (5.1) |

* including bevacizumab.

**Supplementary Table S2. Best overall tumor response in the full analysis set**

| Variables | Vebreltinib group  (*n* = 42) | Control group  (*n* = 39) |
| --- | --- | --- |
| Response, *n* (%) | | |
| Complete response | 1 (2.4) | 1 (2.6) |
| Partial response | 3 (7.1) | 0 (0.0) |
| Stable disease | 20 (47.6) | 12 (30.8) |
| Progressive disease | 14 (33.3) | 18 (46.2) |
| Not assessable | 4 (9.5) | 8 (20.5) |
| Objective response rate* | 4 (9.5) | 1 (2.6) |
| 95% confidence interval for objective response rate | 2.7-22.6 | 0.1-13.5 |

**P* value for comparison of ORR between groups = 0.361. Fisher exact probability test was used for calculation.

**Supplementary Table S3. Treatment-emergent adverse events occurring in at least 5% of patients in the vebreltinib group (*n* = 43)**

| Events, *n* (%) | Grade 1 | Grade 2 | Grade 3 | Grade 4 | Grade 5 | Total |
| --- | --- | --- | --- | --- | --- | --- |
| Any event | 1 (2.3) | 19 (44.2) | 15 (34.9) | 4 (9.3) | 3 (7.0) | 42 (97.7) |
| Epilepsy | 7 (16.3) | 4 (9.3) | 3 (7.0) | 0 (0.0) | 0 (0.0) | 14 (32.6) |
| Vomiting | 10 (23.3) | 4 (9.3) | 0 (0.0) | 0 (0.0) | 0 (0.0) | 14 (32.6) |
| Headache | 6 (14.0) | 3 (7.0) | 4 (9.3) | 0 (0.0) | 0 (0.0) | 13 (30.2) |
| Rash | 5 (11.6) | 6 (14.0) | 0 (0.0) | 0 (0.0) | 0 (0.0) | 11 (25.6) |
| Peripheral edema | 4 (9.3) | 7 (16.3) | 0 (0.0) | 0 (0.0) | 0 (0.0) | 11 (25.6) |
| Alanine aminotransferase increased | 3 (7.0) | 3 (7.0) | 1 (2.3) | 1 (2.3) | 0 (0.0) | 8 (18.6) |
| Hypokalemia | 3 (7.0) | 4 (9.3) | 1 (2.3) | 0 (0.0) | 0 (0.0) | 8 (18.6) |
| Intracranial pressure increased | 0 (0.0) | 7 (16.3) | 1 (2.3) | 0 (0.0) | 0 (0.0) | 8 (18.6) |
| Aspartate aminotransferase increased | 6 (14.0) | 1 (2.3) | 1 (2.3) | 0 (0.0) | 0 (0.0) | 8 (18.6) |
| Constipation | 4 (9.3) | 3 (7.0) | 0 (0.0) | 0 (0.0) | 0 (0.0) | 7 (16.3) |
| Nasopharyngitis | 3 (7.0) | 3 (7.0) | 0 (0.0) | 0 (0.0) | 0 (0.0) | 6 (14.0) |
| Nausea | 3 (7.0) | 3 (7.0) | 0 (0.0) | 0 (0.0) | 0 (0.0) | 6 (14.0) |
| Brain edema | 3 (7.0) | 3 (7.0) | 0 (0.0) | 0 (0.0) | 0 (0.0) | 6 (14.0) |
| Cerebral edema | 1 (2.3) | 3 (7.0) | 2 (4.7) | 0 (0.0) | 0 (0.0) | 6 (14.0) |
| Lethargy | 4 (9.3) | 2 (4.7) | 0 (0.0) | 0 (0.0) | 0 (0.0) | 6 (14.0) |
| Hypoalbuminemia | 4 (9.3) | 0 (0.0) | 1 (2.3) | 0 (0.0) | 0 (0.0) | 5 (11.6) |
| Muscle weakness | 2 (4.7) | 2 (4.7) | 1 (2.3) | 0 (0.0) | 0 (0.0) | 5 (11.6) |
| Urine leukocyte positive | 4 (9.3) | 1 (2.3) | 0 (0.0) | 0 (0.0) | 0 (0.0) | 5 (11.6) |
| Urinary incontinence | 4 (9.3) | 1 (2.3) | 0 (0.0) | 0 (0.0) | 0 (0.0) | 5 (11.6) |
| Blood bilirubin increased | 2 (4.7) | 3 (7.0) | 0 (0.0) | 0 (0.0) | 0 (0.0) | 5 (11.6) |
| Hypoproteinemia | 1 (2.3) | 3 (7.0) | 0 (0.0) | 0 (0.0) | 0 (0.0) | 4 (9.3) |
| Diarrhea | 2 (4.7) | 2 (4.7) | 0 (0.0) | 0 (0.0) | 0 (0.0) | 4 (9.3) |
| Upper respiratory tract infection | 3 (7.0) | 1 (2.3) | 0 (0.0) | 0 (0.0) | 0 (0.0) | 4 (9.3) |
| Limb pain | 1 (2.3) | 3 (7.0) | 0 (0.0) | 0 (0.0) | 0 (0.0) | 4 (9.3) |
| White blood cell count decreased | 2 (4.7) | 1 (2.3) | 0 (0.0) | 0 (0.0) | 0 (0.0) | 3 (7.0) |
| Hypertension | 0 (0.0) | 1 (2.3) | 2 (4.7) | 0 (0.0) | 0 (0.0) | 3 (7.0) |
| Cough | 1 (2.3) | 1 (2.3) | 1 (2.3) | 0 (0.0) | 0 (0.0) | 3 (7.0) |
| Memory impairment | 3 (7.0) | 0 (0.0) | 0 (0.0) | 0 (0.0) | 0 (0.0) | 3 (7.0) |
| Lymphocyte count decreased | 0 (0.0) | 2 (4.7) | 1 (2.3) | 0 (0.0) | 0 (0.0) | 3 (7.0) |
| Anemia | 1 (2.3) | 1 (2.3) | 1 (2.3) | 0 (0.0) | 0 (0.0) | 3 (7.0) |
| Blurred vision | 2 (4.7) | 0 (0.0) | 0 (0.0) | 1 (2.3) | 0 (0.0) | 3 (7.0) |
| Platelet count decreased | 2 (4.7) | 0 (0.0) | 1 (2.3) | 0 (0.0) | 0 (0.0) | 3 (7.0) |
| Altered state of consciousness | 1 (2.3) | 2 (4.7) | 0 (0.0) | 0 (0.0) | 0 (0.0) | 3 (7.0) |
| Tremor | 3 (7.0) | 0 (0.0) | 0 (0.0) | 0 (0.0) | 0 (0.0) | 3 (7.0) |

**Supplementary Table S4. Treatment-emergent adverse events occurring in at least 5% of patients in the control group (*n* = 41)**

| **Events, *n* (%)** | **Grade 1** | **Grade 2** | **Grade 3** | **Grade 4** | **Grade 5** | **Total** |
| --- | --- | --- | --- | --- | --- | --- |
| Any event | 2 (4.9) | 12 (29.3) | 12 (29.3) | 7 (17.1) | 2 (4.9) | 35 (85.4) |
| Vomiting | 6 (14.6) | 8 (19.5) | 0 (0.0) | 0 (0.0) | 0 (0.0) | 14 (34.1) |
| Constipation | 6 (14.6) | 5 (12.2) | 0 (0.0) | 0 (0.0) | 0 (0.0) | 11 (26.8) |
| Epilepsy seizure | 3 (7.3) | 3 (7.3) | 3 (7.3) | 0 (0.0) | 0 (0.0) | 9 (22.0) |
| Nausea | 6 (14.6) | 3 (7.3) | 0 (0.0) | 0 (0.0) | 0 (0.0) | 9 (22.0) |
| Headache | 1 (2.4) | 1 (2.4) | 7 (17.1) | 0 (0.0) | 0 (0.0) | 9 (22.0) |
| White blood cell count decreased | 0 (0.0) | 4 (9.8) | 3 (7.3) | 0 (0.0) | 0 (0.0) | 7 (17.1) |
| Loss of appetite | 6 (14.6) | 0 (0.0) | 0 (0.0) | 0 (0.0) | 0 (0.0) | 6 (14.6) |
| Altered state of consciousness | 0 (0.0) | 4 (9.8) | 0 (0.0) | 1 (2.4) | 0 (0.0) | 5 (12.2) |
| Infectious pneumonia | 0 (0.0) | 2 (4.9) | 0 (0.0) | 2 (4.9) | 0 (0.0) | 4 (9.8) |
| Urinary incontinence | 2 (4.9) | 2 (4.9) | 0 (0.0) | 0 (0.0) | 0 (0.0) | 4 (9.8) |
| Neutrophil count decreased | 0 (0.0) | 2 (4.9) | 2 (4.9) | 0 (0.0) | 0 (0.0) | 4 (9.8) |
| Alanine aminotransferase increased | 2 (4.9) | 1 (2.4) | 0 (0.0) | 0 (0.0) | 0 (0.0) | 3 (7.3) |
| Abnormal liver function | 2 (4.9) | 1 (2.4) | 0 (0.0) | 0 (0.0) | 0 (0.0) | 3 (7.3) |
| Muscle weakness | 1 (2.4) | 2 (4.9) | 0 (0.0) | 0 (0.0) | 0 (0.0) | 3 (7.3) |
| Intracranial pressure increased | 0 (0.0) | 2 (4.9) | 1 (2.4) | 0 (0.0) | 0 (0.0) | 3 (7.3) |
| Anemia | 1 (2.4) | 1 (2.4) | 1 (2.4) | 0 (0.0) | 0 (0.0) | 3 (7.3) |
| Blurred vision | 2 (4.9) | 1 (2.4) | 0 (0.0) | 0 (0.0) | 0 (0.0) | 3 (7.3) |
| Weight loss | 1 (2.4) | 1 (2.4) | 1 (2.4) | 0 (0.0) | 0 (0.0) | 3 (7.3) |
| Platelet count decreased | 0 (0.0) | 2 (4.9) | 0 (0.0) | 1 (2.4) | 0 (0.0) | 3 (7.3) |

**Supplementary Table S5. Treatment-related adverse events occurring in at least 5% and serious adverse events in the vebreltinib group (*n* = 43)**

| Events, *n* (%) | Grade 1 | Grade 2 | Grade 3 | Grade 4 | Grade 5 | Total |
| --- | --- | --- | --- | --- | --- | --- |
| Any treatment-related adverse event | 11 (25.6) | 12 (27.9) | 2 (4.7) | 1 (2.3) | 0 (0.0) | 26 (60.5) |
| Rash | 5 (11.6) | 6 (14.0) | 0 (0.0) | 0 (0.0) | 0 (0.0) | 11 (25.6) |
| Peripheral edema | 3 (7.0) | 3 (7.0) | 0 (0.0) | 0 (0.0) | 0 (0.0) | 6 (14.0) |
| Alanine aminotransferase increased | 2 (4.7) | 1 (2.3) | 1 (2.3) | 1 (2.3) | 0 (0.0) | 5 (11.6) |
| Aspartate aminotransferase increased | 3 (7.0) | 1 (2.3) | 1 (2.3) | 0 (0.0) | 0 (0.0) | 5 (11.6) |
| Vomiting | 4 (9.3) | 0 (0.0) | 0 (0.0) | 0 (0.0) | 0 (0.0) | 4 (9.3) |
| Lymphocyte count decreased | 0 (0.0) | 2 (4.7) | 1 (2.3) | 0 (0.0) | 0 (0.0) | 3 (7.0) |
| Blood bilirubin increased | 2 (4.7) | 1 (2.3) | 0 (0.0) | 0 (0.0) | 0 (0.0) | 3 (7.0) |
| Any SAE | 0 (0.0) | 0 (0.0) | 13 (30.2) | 2 (4.7) | 3 (7.0) | 18 (41.9) |
| Epilepsy seizure | 1 (2.3) | 0 (0.0) | 3 (7.0) | 0 (0.0) | 0 (0.0) | 4 (9.3) |
| Headache | 0 (0.0) | 0 (0.0) | 4 (9.3) | 0 (0.0) | 0 (0.0) | 4 (9.3) |
| Brain edema | 0 (0.0) | 1 (2.3) | 2 (4.7) | 0 (0.0) | 0 (0.0) | 3 (7.0) |
| Hydrocephalus | 0 (0.0) | 0 (0.0) | 1 (2.3) | 1 (2.3) | 0 (0.0) | 2 (4.7) |
| Intracranial pressure increased | 0 (0.0) | 0 (0.0) | 1 (2.3) | 0 (0.0) | 0 (0.0) | 1 (2.3) |
| Dizziness | 0 (0.0) | 0 (0.0) | 1 (2.3) | 0 (0.0) | 0 (0.0) | 1 (2.3) |
| Motor dysfunction | 0 (0.0) | 0 (0.0) | 1 (2.3) | 0 (0.0) | 0 (0.0) | 1 (2.3) |
| Chest discomfort | 0 (0.0) | 0 (0.0) | 1 (2.3) | 0 (0.0) | 0 (0.0) | 1 (2.3) |
| Brain herniation | 0 (0.0) | 0 (0.0) | 0 (0.0) | 0 (0.0) | 1 (2.3) | 1 (2.3) |
| Craniocerebral injury | 0 (0.0) | 0 (0.0) | 1 (2.3) | 0 (0.0) | 0 (0.0) | 1 (2.3) |
| Hypoglycaemia | 0 (0.0) | 0 (0.0) | 0 (0.0) | 1 (2.3) | 0 (0.0) | 1 (2.3) |
| Intervertebral disc protrusion | 0 (0.0) | 0 (0.0) | 1 (2.3) | 0 (0.0) | 0 (0.0) | 1 (2.3) |
| Pneumonitis | 0 (0.0) | 0 (0.0) | 0 (0.0) | 0 (0.0) | 1 (2.3) | 1 (2.3) |
| Drug eruption | 0 (0.0) | 0 (0.0) | 1 (2.3) | 0 (0.0) | 0 (0.0) | 1 (2.3) |
| Vomiting | 0 (0.0) | 1 (2.3) | 0 (0.0) | 0 (0.0) | 0 (0.0) | 1 (2.3) |
| Death | 0 (0.0) | 0 (0.0) | 0 (0.0) | 0 (0.0) | 1 (2.3) | 1 (2.3) |
| Treatment-related SAE | 0 (0.0) | 0 (0.0) | 0 (0.0) | 0 (0.0) | 0 (0.0) | 0 (0.0) |

Abbreviations: SAE, serious adverse event.

**Supplementary Table S6. Treatment-related adverse events occurring in at least 5% and serious adverse events in the control group (*n* = 41)**

| Events, *n* (%) | Grade 1 | Grade 2 | Grade 3 | Grade 4 | Grade 5 | Total |
| --- | --- | --- | --- | --- | --- | --- |
| Any treatment-related adverse event | 8 (19.5) | 7 (17.1) | 3 (7.3) | 2 (4.9) | 0 (0.0) | 20 (48.8) |
| Vomiting | 6 (14.6) | 6 (14.6) | 0 (0.0) | 0 (0.0) | 0 (0.0) | 12 (29.3) |
| Nausea | 5 (12.2) | 2 (4.9) | 0 (0.0) | 0 (0.0) | 0 (0.0) | 7 (17.1) |
| White blood cell count decreased | 0 (0.0) | 3 (7.3) | 3 (7.3) | 0 (0.0) | 0 (0.0) | 6 (14.6) |
| Constipation | 4 (9.8) | 2 (4.9) | 0 (0.0) | 0 (0.0) | 0 (0.0) | 6 (14.6) |
| Appetite decreased | 4 (9.8) | 0 (0.0) | 0 (0.0) | 0 (0.0) | 0 (0.0) | 4 (9.8) |
| Neutrophil count decreased | 0 (0.0) | 2 (4.9) | 2 (4.9) | 0 (0.0) | 0 (0.0) | 4 (9.8) |
| Any SAE | 0 (0.0) | 0 (0.0) | 9 (22.0) | 2 (4.9) | 2 (4.9) | 13 (31.7) |
| Headache | 0 (0.0) | 0 (0.0) | 5 (12.2) | 0 (0.0) | 0 (0.0) | 5 (12.2) |
| Epilepsy seizure | 0 (0.0) | 0 (0.0) | 3 (7.3) | 0 (0.0) | 0 (0.0) | 3 (7.3) |
| Intracranial pressure increased | 0 (0.0) | 0 (0.0) | 1 (2.4) | 0 (0.0) | 0 (0.0) | 1 (2.4) |
| Coma | 0 (0.0) | 0 (0.0) | 0 (0.0) | 1 (2.4) | 0 (0.0) | 1 (2.4) |
| Hypersomnia | 0 (0.0) | 0 (0.0) | 1 (2.4) | 0 (0.0) | 0 (0.0) | 1 (2.4) |
| Pyrexia | 0 (0.0) | 0 (0.0) | 1 (2.4) | 0 (0.0) | 0 (0.0) | 1 (2.4) |
| Brain herniation | 0 (0.0) | 0 (0.0) | 0 (0.0) | 0 (0.0) | 1 (2.4) | 1 (2.4) |
| Pneumonitis | 0 (0.0) | 0 (0.0) | 0 (0.0) | 1 (2.4) | 0 (0.0) | 1 (2.4) |
| White blood cell count decreased | 0 (0.0) | 0 (0.0) | 1 (2.4) | 0 (0.0) | 0 (0.0) | 1 (2.4) |
| Death | 0 (0.0) | 0 (0.0) | 0 (0.0) | 0 (0.0) | 1 (2.4) | 1 (2.4) |
| Treatment-related SAE | 0 (0.0) | 0 (0.0) | 1 (2.4) | 0 (0.0) | 0 (0.0) | 1 (2.4) |

Abbreviations: SAE, serious adverse event.

**Supplementary Figures**





**Supplementary Figure S1. Kaplan-Meier curves in the intention-to-treat population (*n* = 84). (A)** Overall survival. **(B)** progression-free survival. Abbreviations: OS, overall survival; HR, hazard ratio; CI, confidence interval; *n*.censor, number of censoring; PFS, progression-free survival.


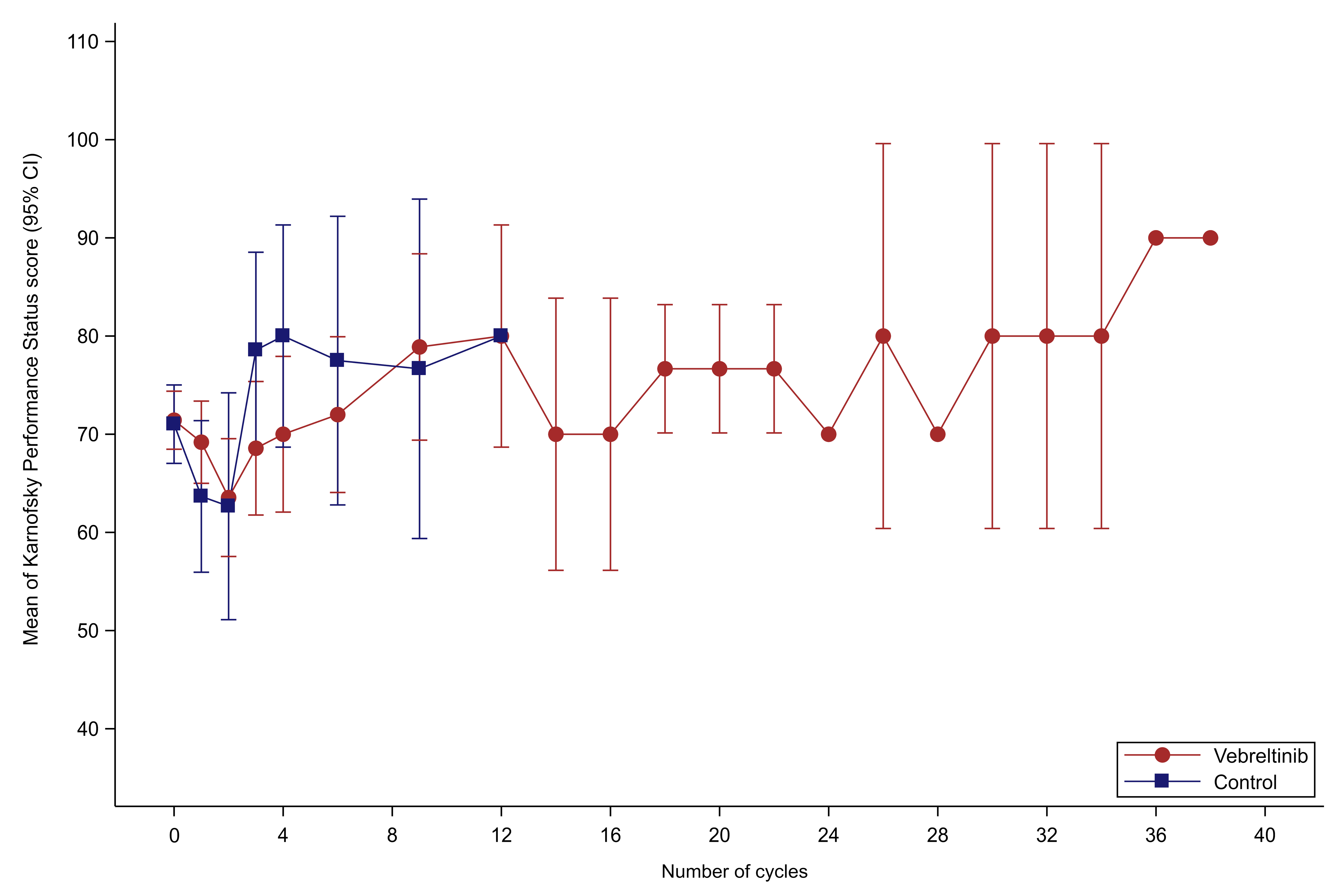


**Supplementary Figure S2. Changes in Karnofsky Performance Status over time in the full analysis set.** Abbreviations: CI, confidence interval.


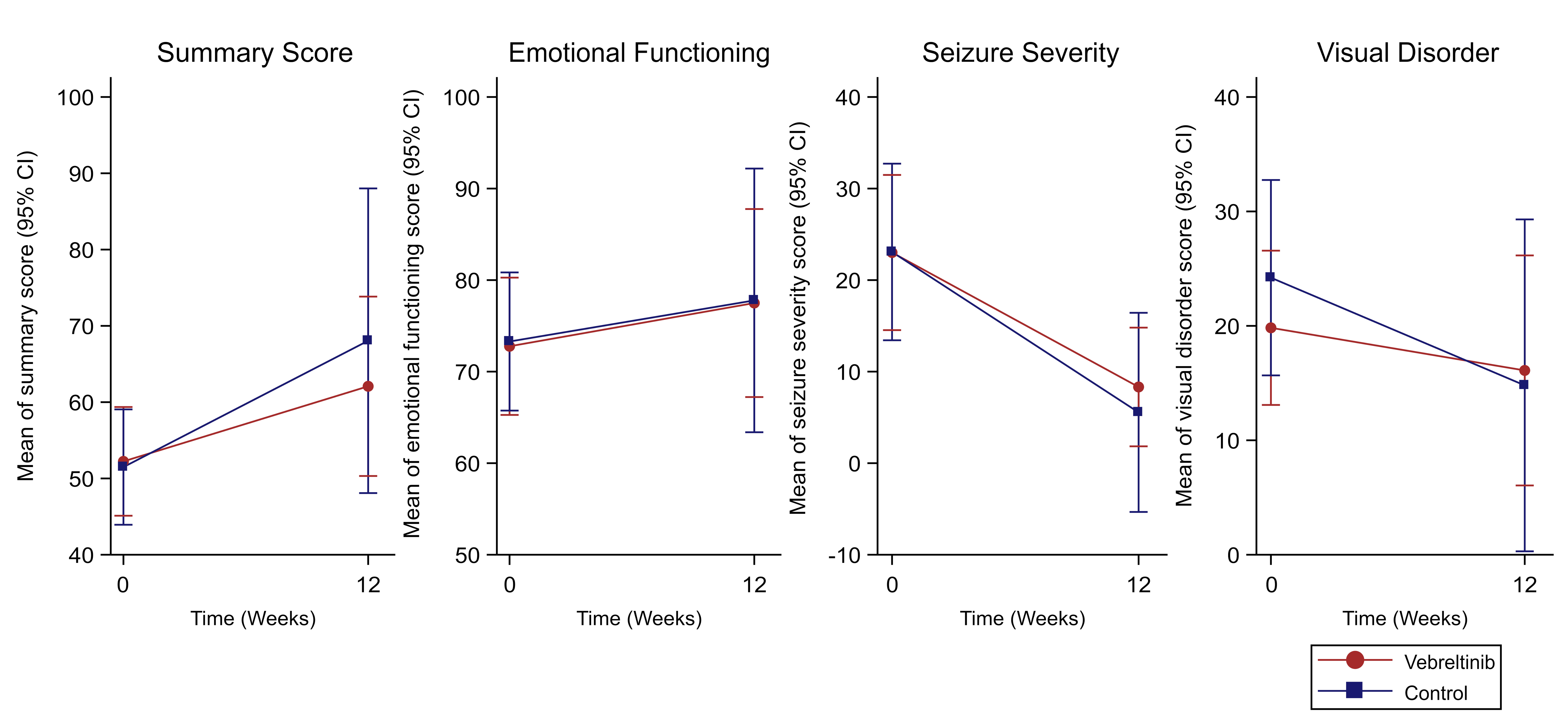


**Supplementary Figure S3. Longitudinal quality-of-life scores in the full analysis set. (A)** QLQ-C30 summary score. **(B)** Emotional functioning score as assessed by the QLQ-C30. **(C)** Seizure severity score as assessed by the QLQ-BN20. **(D)** Visual disorder score as assessed by the QLQ-BN20. Abbreviations: CI, confidence interval; QLQ-C30, Quality of Life Questionnaire Core 30; QLQ-BN20, Quality of Life Questionnaire Brain Cancer Module 20.


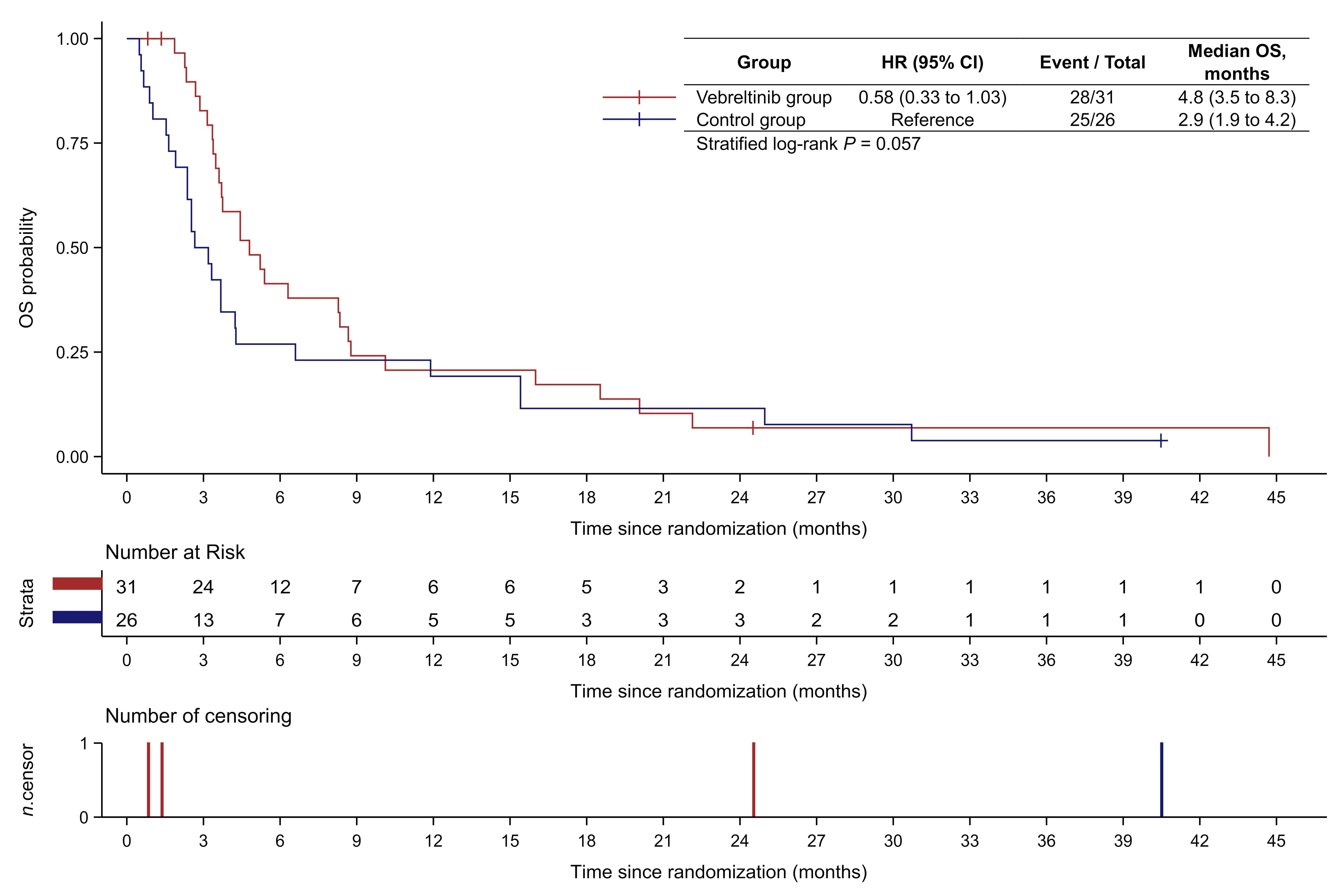


**Supplementary Figure S4. Overall survival in patients with baseline tumor diameter >3.0 cm.**

Abbreviations: OS, overall survival; HR, hazard ratio; CI, confidence interval; *n*.censor, number of censoring.


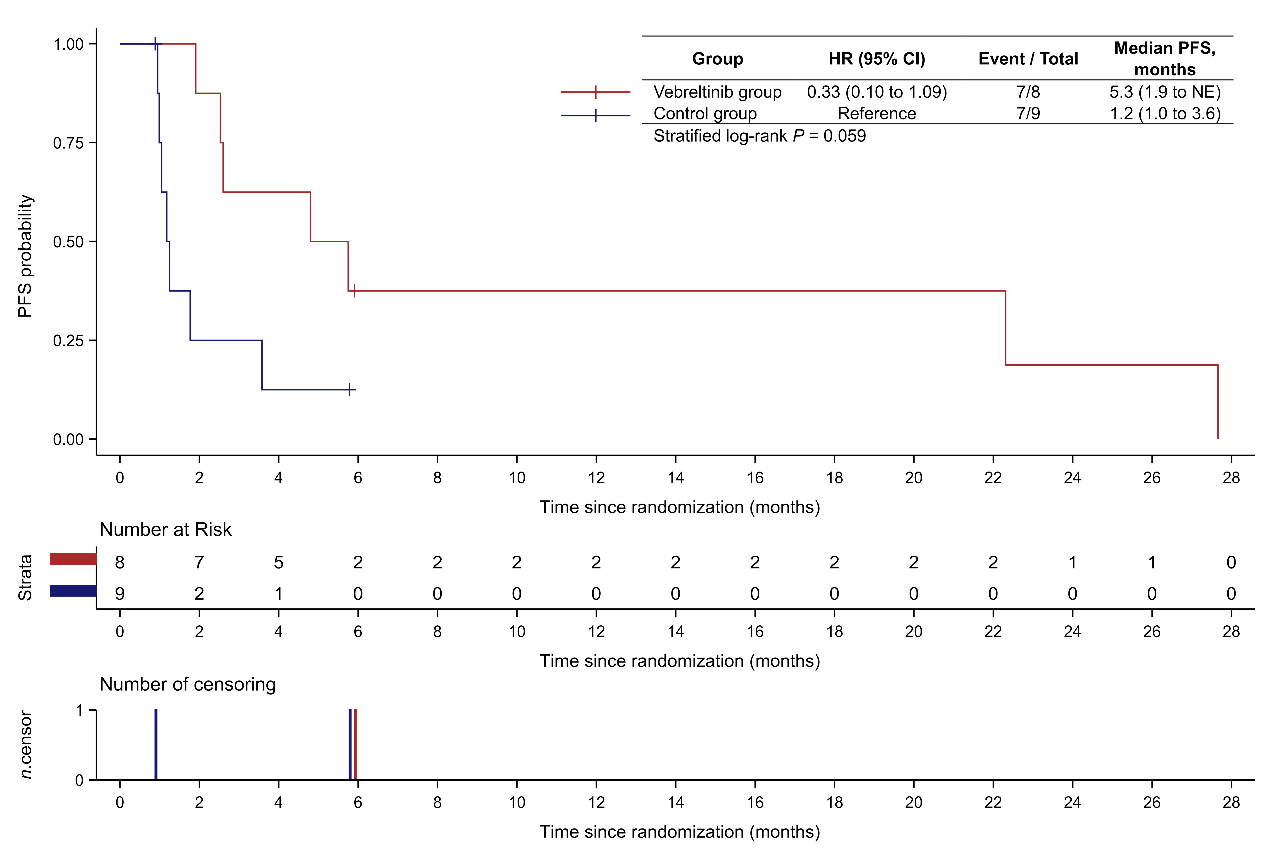


**Supplementary Figure S5. Progression-free survival in patients with baseline tumor diameter ≤3.0 cm.** Abbreviations: PFS, progression-free survival; HR, hazard ratio; CI, confidence interval; NE, not estimable; *n*.censor, number of censoring


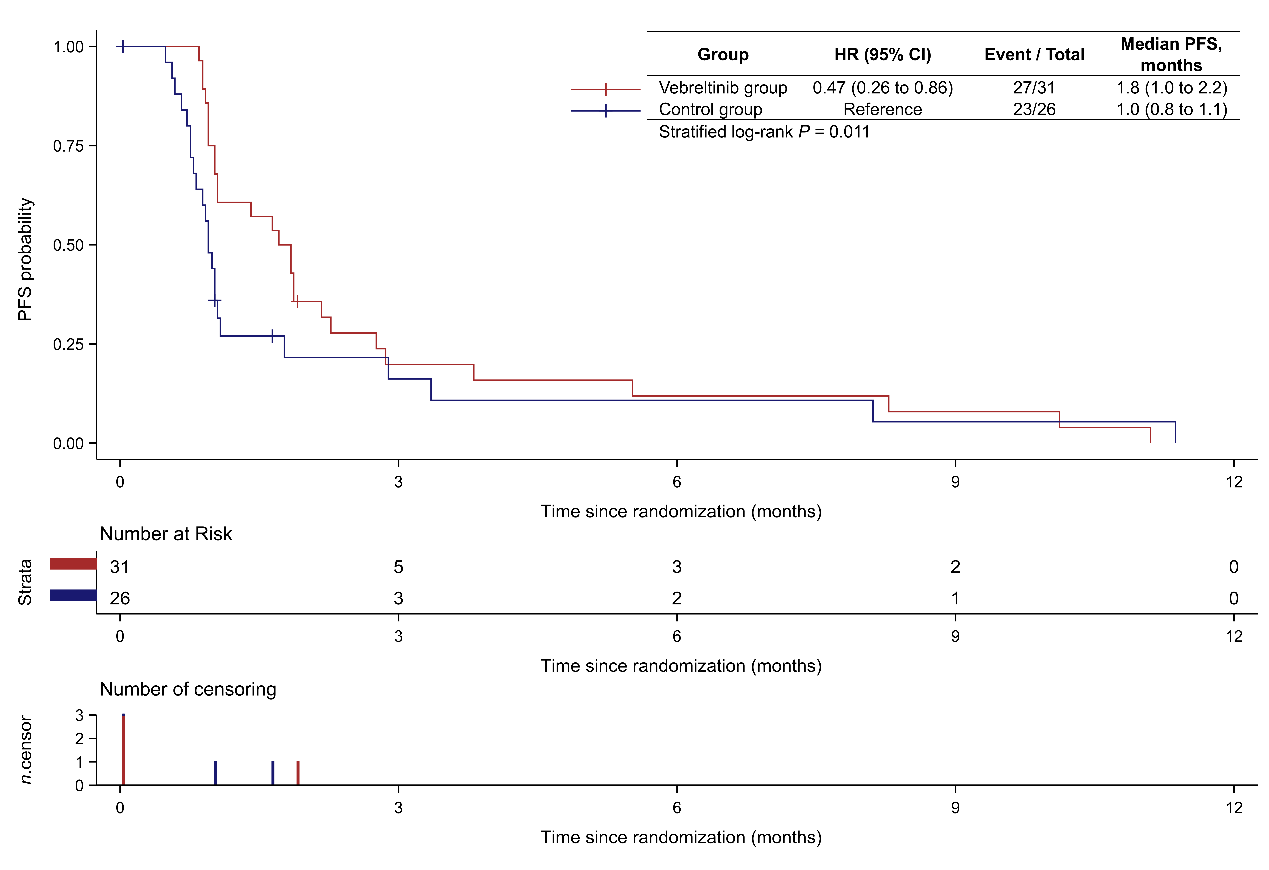


**Supplementary Figure S6. Progression-free survival in patients with baseline tumor diameter >3.0 cm.** Abbreviations: PFS, progression-free survival; HR, hazard ratio,CI, confidence interval; *n*.censor, number of censoring.


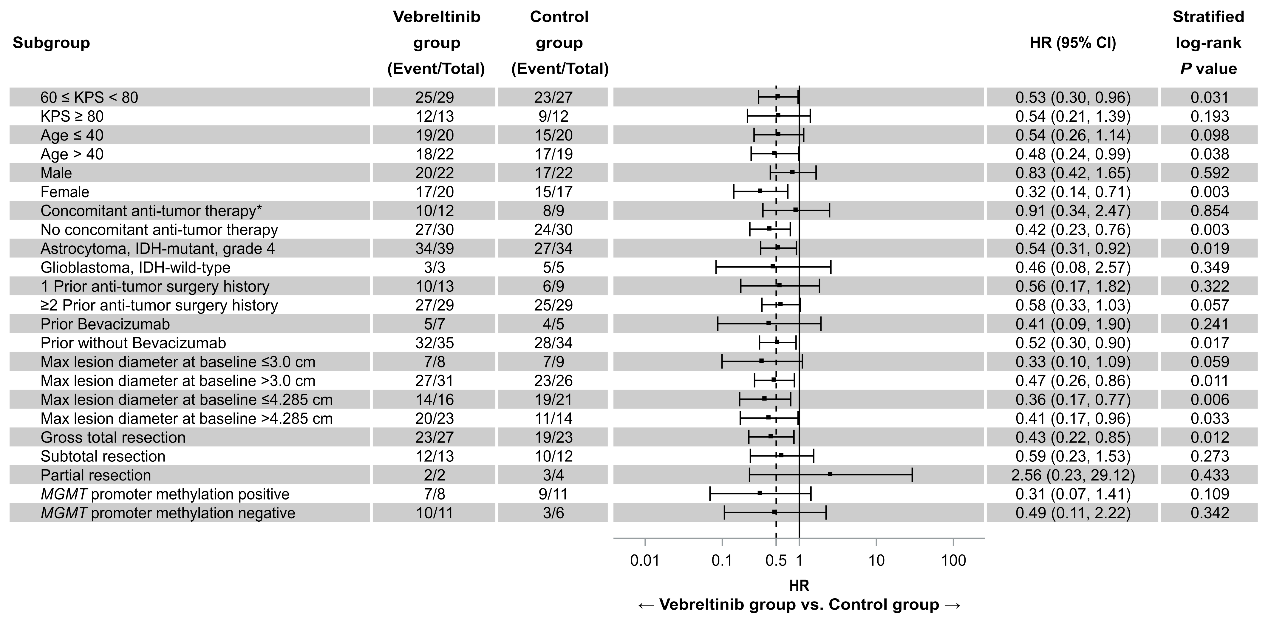


**Supplementary Figure S7. Subgroup analysis of progression-free survival.** Abbreviations: KPS, Karnofsky Performance Status; *IDH,* isocitrate dehydrogenase; max, maximum; *MGMT*, *O^6^-methylguanine-DNA methyltransferase*; HR, hazard ratio; CI, confidence interval. *Concomitant anti-tumor therapy refers to anti-tumor treatment with non-study drugs administered during the survival follow-up period. Note: 4.285 cm was the median baseline value of maximum lesion diameter.

**References:**

1. Louis, D.N., et al., *The 2016 World Health Organization Classification of Tumors of the Central Nervous System: a summary.* Acta Neuropathol, 2016. **131**(6): p. 803-20.

2. Park, Y.W., et al., *The 2021 WHO Classification for Gliomas and Implications on Imaging Diagnosis: Part 1-Key Points of the Fifth Edition and Summary of Imaging Findings on Adult-Type Diffuse Gliomas.* J Magn Reson Imaging, 2023. **58**(3): p. 677-689.
