# Supplementary figures and images for: Vebreltinib for Previously Treated Astrocytoma, IDH-Mutant, Grade 4, and Glioblastoma, IDH Wild-Type with PTPRZ1–MET Fusion Gene: A Multicenter, Phase III Randomized, Open-Label Trial
Source: Cancer Commun (Lond). 2026 Mar 11;46:0019. doi: 10.34133/cancomm.0019 (PMC12976379; doi:10.34133/cancomm.0019)

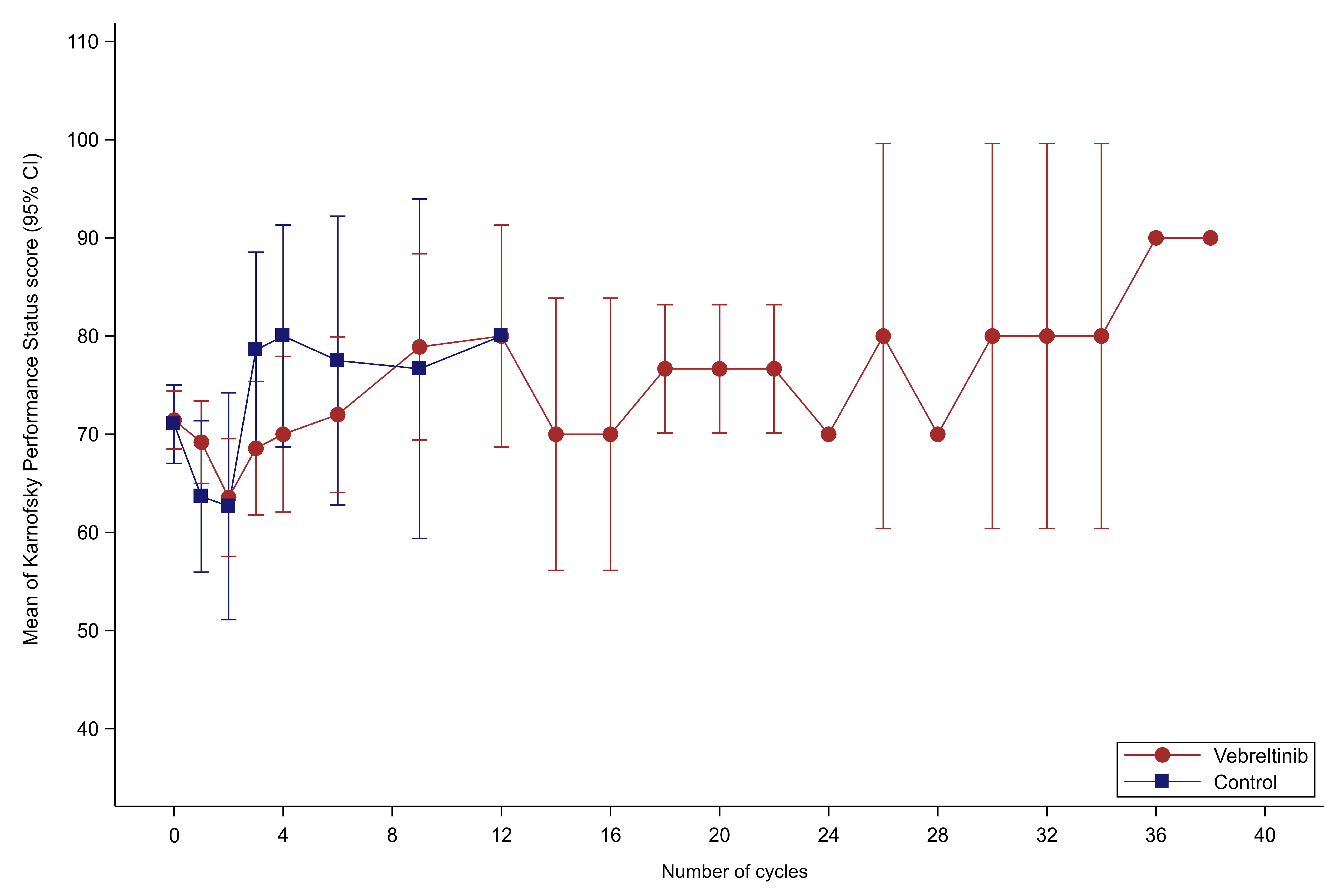

Supplement: Supplementary 1 — Tables S1 to S6 Figs. S1 to S7 [file cancomm.0019.f1.zip › Figure S2-R3.tif]

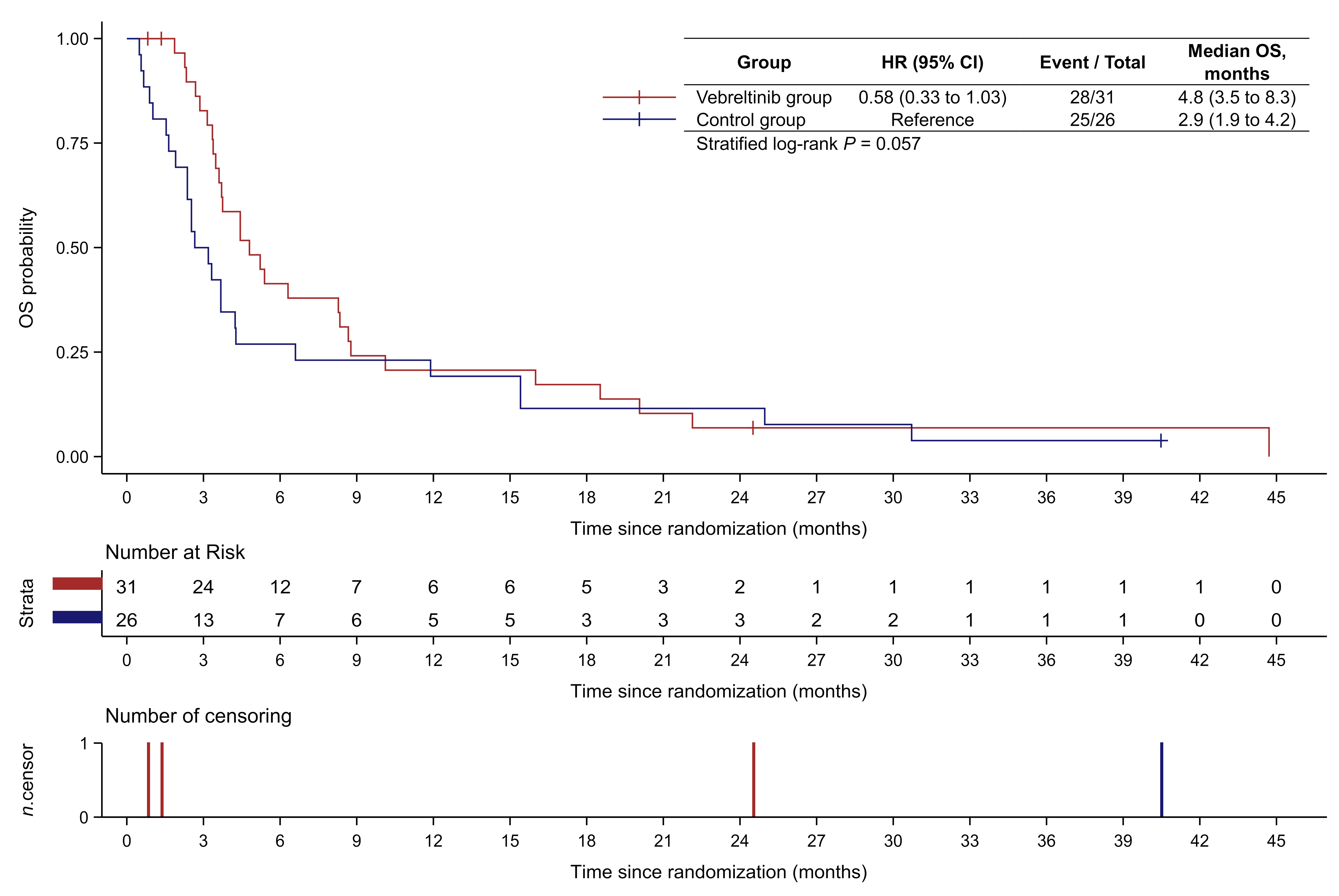

Supplement: Supplementary 1 — Tables S1 to S6 Figs. S1 to S7 [file cancomm.0019.f1.zip › Figure S4-R3.tif]

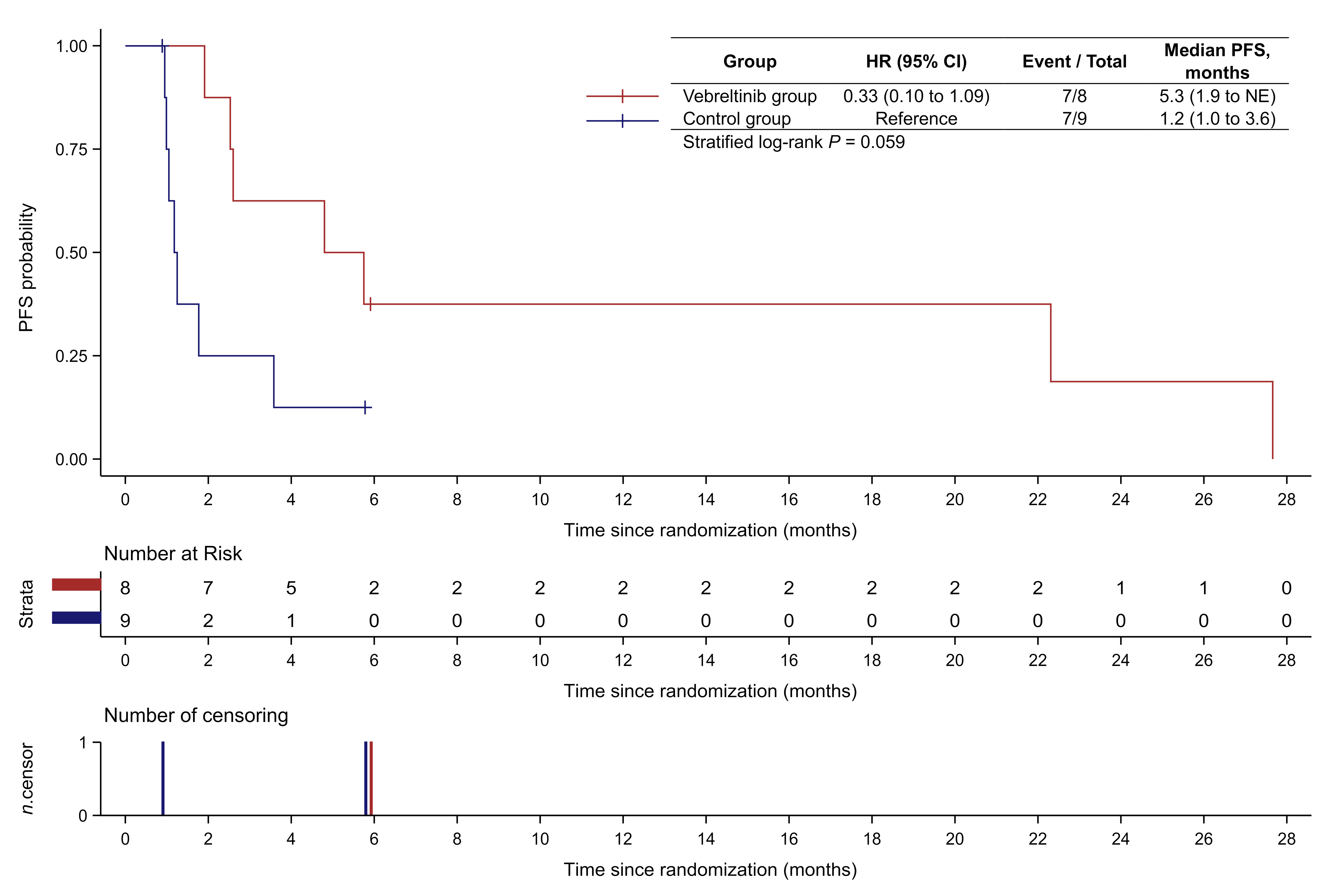

Supplement: Supplementary 1 — Tables S1 to S6 Figs. S1 to S7 [file cancomm.0019.f1.zip › Figure S5-R3.tif]

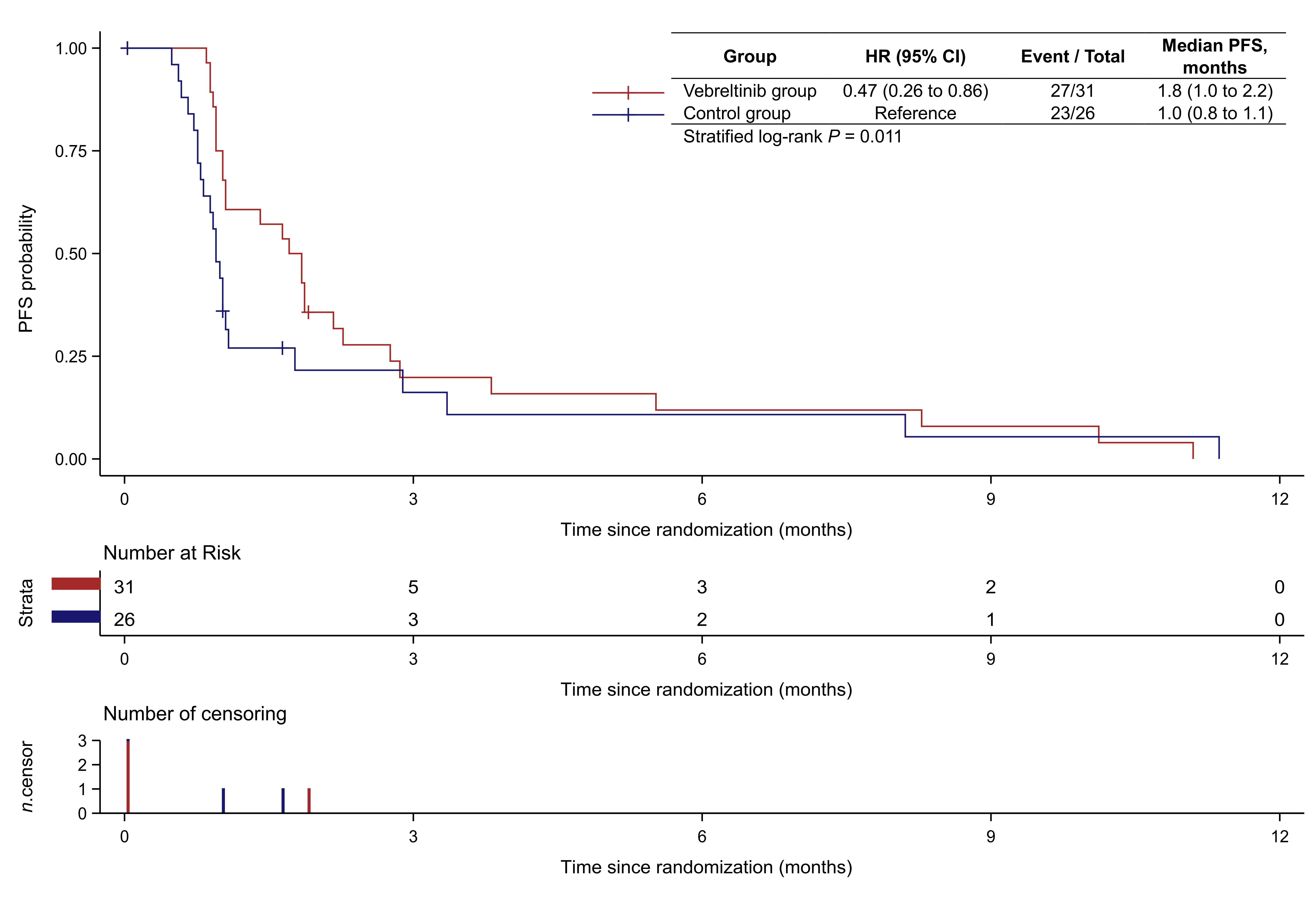

Supplement: Supplementary 1 — Tables S1 to S6 Figs. S1 to S7 [file cancomm.0019.f1.zip › Figure S6-R3.tif]

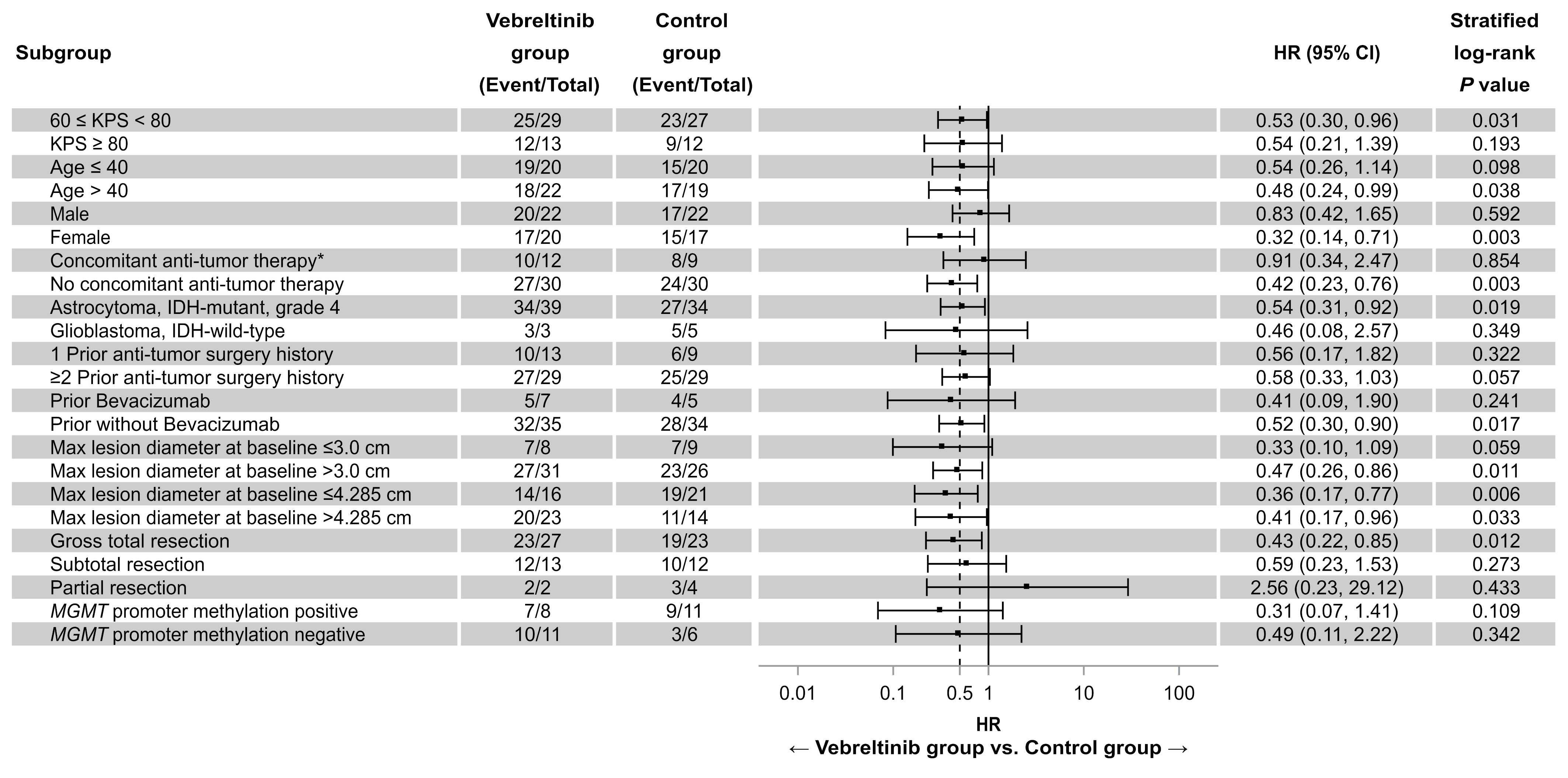

Supplement: Supplementary 1 — Tables S1 to S6 Figs. S1 to S7 [file cancomm.0019.f1.zip › Figure S7-R3.tif]
